# Potassium Hexacyanoferrate (III)-Catalyzed Dimerization of Hydroxystilbene: Biomimetic Synthesis of Indane Stilbene Dimers

**DOI:** 10.3390/molecules201219872

**Published:** 2015-12-18

**Authors:** Jing-Shan Xie, Jin Wen, Xian-Fen Wang, Jian-Qiao Zhang, Ji-Fa Zhang, Yu-Long Kang, You-Wei Hui, Wen-Sheng Zheng, Chun-Suo Yao

**Affiliations:** 1 State Key Laboratory of Bioactive Substance and Function of Natural Medicines, Institute of Materia Medica, Chinese Academy of Medical Sciences & Peking Union Medical College, Beijing 100050, China; jingshan508@126.com (J.-S.X.); wxf312936046@163.com (X.-F.W.); jqzhang@imm.ac.cn (J.-Q.Z.); jifa17@163.com (J.-F.Z.); kangyulongzyy@imm.ac.cn (Y.-L.K.); 2 School of Chemical Engineering, Northwest University, Xi’an 710069, China; youweixi163@163.com; 3 Chinese Pharmaceutical Association, Beijing 100022, China; Jin_ann1463@163.com

**Keywords:** potassium hexacyanoferrate (III), indane stilbene dimer, biomimetic synthesis, hydroxystilbene

## Abstract

Using potassium hexacyanoferrate (III)–sodium acetate as oxidant, the oxidative coupling reaction of isorhapontigenin and resveratrol in aqueous acetone resulted in the isolation of three new indane dimers **4**, **6**, and **7**, together with six known stilbene dimers. Indane dimer **5** was obtained for the first time by direct transformation from isorhapontigenin. The structures and relative configurations of the dimers were elucidated using spectral analysis, and their possible formation mechanisms were discussed. The results indicate that this reaction could be used as a convenient method for the semi-synthesis of indane dimers because of the mild conditions and simple reaction products.

## 1. Introduction

**Figure 1:**
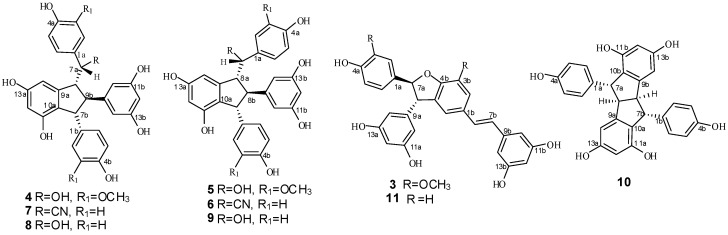
Structures of compounds **3**–**11**.

Stilbene dimers with indane skeletons possess a wide range of biological activity [1,2] and novel structures which are difficult to achieve by common organic reactions on account of their intricate architectures and chiral centers. As many of these compounds are exclusively obtained by extraction from natural sources, the studies of biological properties are limited by their extreme scarcity. This has made the synthesis of stilbene dimers, especially indane dimers a popular research topic [3]. Indane stilbene dimers such as quadragularin A, pallidol, ampelopsin F, paucifloral F, ampelopsin D, and caraphenol C have been successfully synthesized [4,5,6,7,8,9,10]. However, the complexity of the synthetic routes has hindered further studies on these indane derivatives, and the search for simple and convenient synthetic routes to obtain abundant samples is of significant interest. As a conventional inorganic one-electron oxidant, potassium hexacyanoferrate (III) (K_3_Fe(CN)_6_) has been reported to generate resveratrol *trans*-dehydrodimer, ε-viniferin and indane dimers in the oxidative coupling reaction of resveratrol (**1**) [11,12]. However, a detailed study of this reaction has yet to be reported. In our previous paper [13], we reported that the transformation of isorhapontigenin (**2**) with K_3_Fe(CN)_6_/sodium acetate (NaOAc) as oxidant yielded shegansu B (**3**) as the major product peak (65.2%) and another peak (about 10%) according to high-performance liquid chromatography (HPLC). Further investigation showed that the latter peak comprised two indane dimers, **4** and **5**, which indicates that the reaction is amenable for the formation of indane dimers. To substantiate this hypothesis, studies on the oxidative coupling reaction of resveratrol employing the same oxidants were conducted, resulting in the isolation of five resveratrol indane dimers **6**–**10**, and the benzofuran derivative resveratrol *trans*-dehydrodimer **11** (Figure 1). Among the dimers, **4**, **6** and **7** are new indane dimers. This paper reports on the oxidative coupling of **1** and **2** in aqueous acetone with K_3_Fe(CN)_6_/NaOAc as oxidant, the isolation and structural identification of the products, and the discussion of the mechanisms of formation of all the products.

## 2. Results and Discussion

### 2.1. Treatment of ***2*** with Potassium Hexacyanoferrate (III)/Sodium Acetate

**Figure 2:**
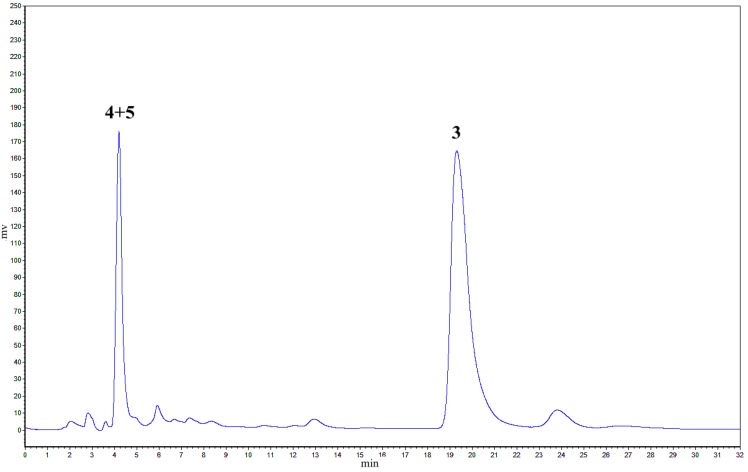
Analysis of isorhapontigenin oxidation products by K_3_Fe(CN)_6_/NaOAc (32% CH_3_CN/H_2_O, λ = 230 nm, 1 mL/min).

As reported in a previous paper [13], the oxidative coupling reaction of **2** in aqueous acetone using K_3_Fe(CN)_6_/NaOAc as oxidant at room temperature generated a major product peak **3** in 65.2% yield and a peak of another product obtained in 10% yield with a retention time of 4.2 min in the HPLC chromatogram (Figure 2).

This finding indicates that the reaction mainly produced two types of products as compared with the complex products seen in the common oxidative coupling reactions of hydroxylstilbene [13,14,15,16]. Furthermore, several reports of K_3_Fe(CN)_6_ molecules catalyzing the oxidative coupling reaction of hydroxystilbene can be found in the literature [11,12]. However, to the best of our knowledge, a detailed investigation on this reaction, especially for the K_3_Fe(CN)_6_/NaOAc oxidant system, has yet to be reported. In this study, isorhapontigenin was treated with K_3_Fe(CN)_6_/NaOAc in aqueous acetone at room temperature, followed by silica gel column chromatography, preparative HPLC, and semi-preparative HPLC to obtain a major product **3** in 52.2% yield, as well as two indane dimers **4** and **5** (Figure 1) in 6.0% and 3.3% yield, respectively. Among these dimers, **5** possesses the same structure as the natural product gnetuhainin I [17], and compound **4**, an isomer of **5**, is a new isorhapontigenin dimer. These indane dimers were obtained for the first time by direct transformation from isorhapontigenin. The results imply that these reaction conditions which led to a total yield of about 10% for the indane dimers should be beneficial to the formation of carbon-carbon bonds.

### 2.2. Treatment of ***1*** with Potassium Hexacyanoferrate (III)/Sodium Acetate

**Figure 3:**
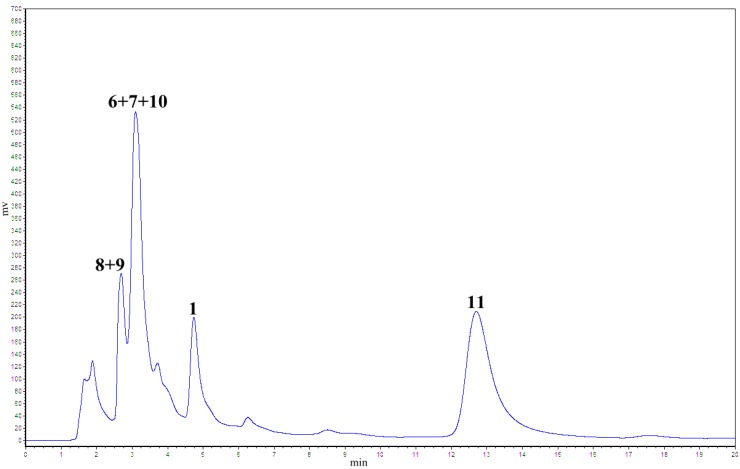
Analysis of resveratrol oxidation products by K_3_Fe(CN)_6_/NaOAc (52% MeOH/H_2_O, λ = 230 nm, 1 mL/min).

To substantiate the above hypothesis, we conducted a further study on the oxidative coupling reaction of resveratrol catalyzed by K_3_Fe(CN)_6_/NaOAc in aqueous acetone under reflux for 60 h. This approach was used because when resveratrol was treated at room temperature for one week, only small amounts of products were observed, and large amounts of unchanged resveratrol were recovered. Under the optimized conditions, two major products were observed on TLC plates, whereas other than the major products, several peaks ascribed to indane isomers of resveratrol dimer on the basis of previous results, appeared in the HPLC chromatogram (Figure 3). Therefore, by application of the above conditions, the oxidative coupling reaction of 1000 mg resveratrol, combined with silica gel column chromatography (CC), preparative HPLC, and semi-preparative HPLC, led to the isolation of six resveratrol dimers: one major benzofuran product **11** (21.1% yield) and five indane dimers **6** (4.9% yield), **7** (5.9%), **8** (1.3%), **9** (2.1%), and **10** (0.8%, Figure 1). Among these dimers, **6** and **7** are new indane stilbene dimers that are substituted by a cyano group. Dimers **8**, **9**, **10**, and **11** possess the same structures as the natural products leachianol G, leachianol F, pallidol, and resveratrol *trans*-dehydrodimer. The total yield of about 15% for indane products indicates that the indane dimer is one of the major products in this reaction. 

### 2.3. Structural Identification of New Dimers

**Figure 4:**
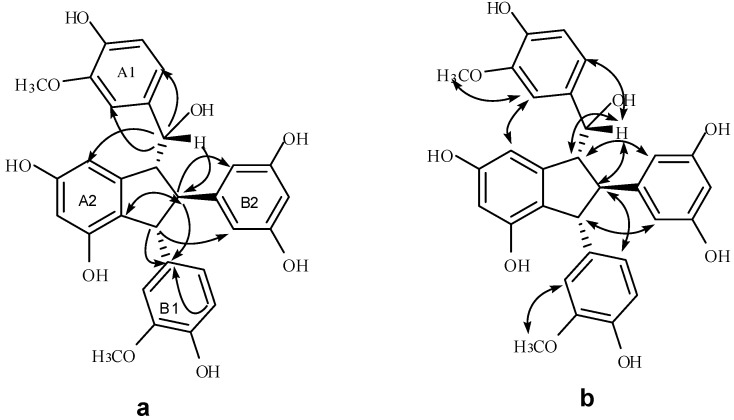
The significant HMBC (**a**) and NOESY (**b**) interactions of **4**.

Compound **4** was isolated as a brown amorphous powder. The corresponding negative ion HR-ESI-MS (Figure S8) peak at *m/z* 531.1662 [M − H]^−^ (calcd. for C_30_H_27_O_9_, 531.1661) showed the molecular formula of C_30_H_28_O_9_, which together with the ^1^H- and ^13^C-NMR spectral data, suggests that **4** should be an isorhapontigenin dimer. The IR spectrum (Figure S10) displays the presence of hydroxyls (3360 cm^−1^) and aromatic groups (1605 and 1516 cm^−1^). The ^1^H-NMR spectrum (Figure S1, Table 1) shows two ABX systems for rings A1 and B1 at δ_H_ 6.82 (1H, d, 1.8), 6.61 (1H, d, 7.8), 6.57 (1H, dd, 7.8, 1.8), and 6.37 (1H, d, 1.8), 6.55 (1H, d, 8.4), and 6.30 (1H, dd, 8.4, 1.8), one AB_2_ system at δ_H_ 6.02 (1H, t, 2.4) and 6.03 (2H, d, 2.4) for ring B2, two *meta*-coupled proton signals at δ_H_ 6.08 (1H, d, 1.8) and 6.07 (1H, brs) for ring A2, four multi-coupled aliphatic protons at δ_H_ 4.48 (1H, d, 7.2), 4.08 (1H, d, 4.8), 3.49 (1H, t, 6.0), and 3.17 (1H, t, 5.4) ppm, and two methoxyl singlets at δ_H_ 3.61 (3H, s), and 3.65 (3H, s). The ^13^C-NMR spectrum (Figure S2, Table 1) of **4** reveals the presence of four aliphatic carbons at δ_C_ 78.0, 61.8, 60.0, and 57.4 ppm, as well as 24 aromatic carbons and two methoxy carbons. The aliphatic carbon at δ_C_ 78.0 is due to an alcohol carbon. This group of evidence indicates that compound **4** possesses a similar indane skeleton as **5**, as reported in the literature [17]. In addition, downfield shifts of H-2a, H-6a, H-8b, and H-10(14)b and the corresponding upfield shift of H-14a caused by the anisotropic effect of the aromatic ring in comparison to those of **5** proved that **4** should be an 7-epimer of **5** [18]. In the HMBC spectrum of **4** (Figure 4), the correlations among H-2a, H-6a, H-14a and C-7a, which is attached to the hydroxyl group, indicates that C-7a is excluded from the additional ring. The correlations between H-7b, H-8b, H-5b and C-1b verify that the B1 ring should be connected at C-7b. Comparison of the spectral data with those of **5**, as well as the analysis of COSY, HMBC and HSQC correlations (Figures S4–S6), determines the planar structure of **4** as shown in Figure 1. The stereochemistry of **4** was determined by analysis of NOESY spectrum (Figure S7 and Figure 4), in which strong NOEs between H-10(14)b with H-7b and H-8a suggests a *trans* orientation between H-7b and H-8b as well as between H-8b and H-8a. The NOE interactions between H-7a and H-8b revealed a *cis* relationship of H-7a and H-8b. Accordingly, the structure of **4** was determined as shown in Figure 1.

**Table 1:** ^1^H- and ^13^C-NMR spectroscopic data of **4** and **5** *.

Position	4		5	
	δ_C_	δ_H_	δ_C_	δ_H_
1a	135.5s		136.5s	
2a	112.4d	6.82 (d, 1.8)	112.4d	6.37 (d, 1.8)
3a	148.5s		148.7d	
4a	146.9s		146.7s	
5a	115.3d	6.61 (d, 7.8)	115.74d	6.59 (d, 7.8),
6a	121.1d	6.57 (dd, 7.8, 1.8)	120.94d	6.39 (dd, 7.8, 1.8)
7a	78.0d	4.48 (d, 7.2)	78.7d	4.35 (d, 9.0)
8a	61.8d	3.49 (t, 6.0)	62.0d	3.29 (overlap)
9a	148.7s		149.5s	
10a	123.9s		123.0s	
11a	155.4s		155.1s	
12a	102.6	6.08 (d, 1.8)	102.6d	6.19 (d, 1.8)
13a	158.8s		159.2s	
14a	106.0d	6.07 (br s)	106.30d	6.58 (d, 1.8)
1b	139.0s		138.8s	
2b	112.0d	6.37 (d, 1.8)	111.9d	6.49 (d, 1.8)
3b	147.4s		148.5s	
4b	145.5s		145.6s	
5b	115.6d	6.55 (d, 8.4)	115.69d	6.62 (d, 7.8)
6b	120.0d	6.30 (dd, 8.4, 1.8)	120.93d	6.35 (dd, 7.8, 1.8)
7b	57.4d	4.08 (d, 4.8)	56.4d	4.14 (d, 3.0)
8b	60.0d	3.17 (t, 5.4)	60.2d	2.74 (t, 3.0)
9b	150.7s		151.3s	
10(14)b	106.9d	6.03 (d, 2.4)	106.33d	5.75 (d, 2.4)
11(13)b	159.4s		159.3s	
12b	101.3d	6.02 (t, 2.4)	101.2d	5.96 (t, 2.4)
3a-OCH_3_	56.3q	3.61 (s)	56.3q	3.58 (s)
3b-OCH_3_	56.2q	3.65 (s)	56.1q	3.67 (s)

* Data (δ) were measured in CD_3_OD for ^1^H at 600 MHz, and for ^13^C at 150 MHz, The assignments were based on DEPT, ^1^H-^1^H COSY, HSQC, HMBC, and NOESY experiments, respectively.

**Figure 5:**
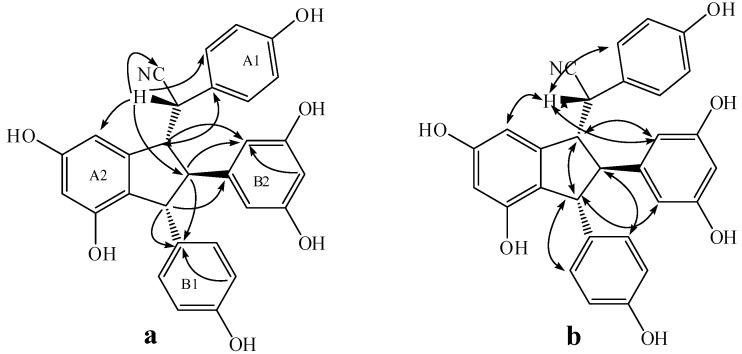
The key HMBC (**a**) and NOESY (**b**) correlations of **6**.

Compound **6** was obtained as a light gray amorphous powder. The corresponding positive ion HR-ESI-MS (Figure S17) peak at *m/z* 504.1409 [M + Na]^+^ (calcd. for C_29_H_23_NO_6_Na, 504.1418) yield the molecular formula of C_29_H_23_NO_6_, which indicates that **6** should be a resveratrol dimer derivative. The IR spectrum (Figure S19) displayed the presence of hydroxyls (3393.9 cm^−1^), cyano group (2248.6 cm^−1^), and aromatic rings (1610.6, 1513.2, 1468.4 cm^−1^). The ^1^H-NMR spectrum (Table 2) shows two A_2_B_2_ systems for ring A1 and B1 at δ_H_ 6.89 (2H, d, 8.5), 6.79 (2H, d, 8.5), 6.87 (2H, d, 8.5), and 6.76 (2H, d, 8.5), one AB_2_ system for ring B2 at δ_H_ 5.96 (2H, d, 2.0) and 6.17 (1H, t, 2.0), two *meta*-coupled proton signals for ring A2 at δ_H_ 6.38 (1H, d, 2.0) and 6.57 (1H, d, 2.0), and four multi-coupled aliphatic proton signals at δ_H_ 4.28 (1H, d, 4.0), 3.76 (1H, d, 8.5), 3.52 (1H, dd, 8.5, 4.0), and 3.01 (1H, t, 4.0). C_29_H_23_NO_6_, the quaternary carbon signal at δ_C_ 120.33 in the ^13^C-NMR data (Figure S12, Table 2) of **6** suggests the presence of a cyano group in the structure of **6**. Accordingly, **6** was assumed to contain a leachianol G skeleton with a cyano group replacing a hydroxyl group [19]. In addition to a cyano group, 22 carbon signals in the ^13^C-NMR representing 28 carbons further support this hypothesis. In the HMBC spectrum (Figure S15, Figure 5), the interactions between H-7a and C-2(6)a, C-14a indicate that ring A1 is located at C-7; the interactions among H-8b, H-12b, and C-10(14)b substantiate that ring B2 is connected at C-8b. Similarly, the correlations of the three proton signals of H-7b, H-8b, and H-3(5)b with C-1b reveal that ring B1 is connected at C-7b. Moreover, in the NOESY spectrum of **6** (Figure S16, Figure 5), the interactions between H-8a and H-7b, H-10(14)b, as well as between H-8b and H-2(6)b suggest a *cis*-configuration among H-8a, H-7b and ring B2. The interactions between H-7a and H-14a indicate that H-7a should be located near H-14a, and that ring A1 is located near ring B_2_. Therefore, the structure of **6** is characterized as shown in Figure 1.

**Figure 6:**
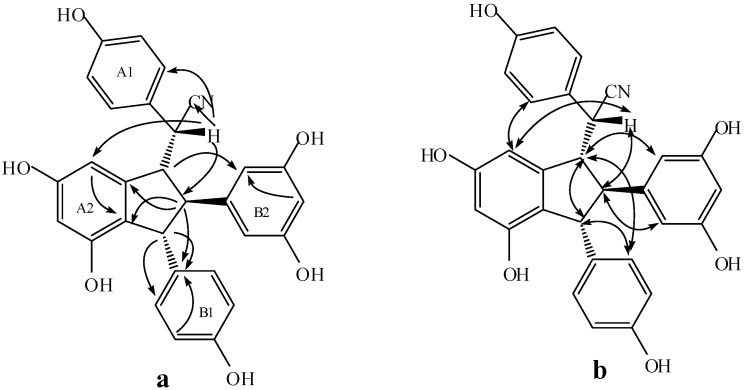
The key HMBC (**a**) and NOESY (**b**) correlations of **7**.

Compound **7** was obtained as a light brown amorphous powder. The corresponding positive ion HR-ESI-MS (Figure S26) peak at *m/z* 504.1431 [M + Na]^+^ (calcd. for C_29_H_23_NO_6_Na, 504.1418) corresponds to the molecular formula of C_29_H_23_NO_6_. The IR spectrum (Figure S28) revealed the presence of hydroxyls (3337.0 cm^−1^), a cyano group (2247.8 cm^−1^), and aromatic rings (1610.9, 1513.5, 1468.2 cm^−1^). The ^1^H-NMR spectrum (Figure S20, Table 2) shows two A_2_B_2_ systems for rings A1 and B1, one AB_2_ system for ring B2, two *meta*-coupled proton signals for ring A2, and four aliphatic proton signals at δ_H_ 4.27 (1H, d, 4.5), 3.96 (1H, d, 8.1), 3.58 (1H, dd, 8.1, 4.5), and 3.26 (1H, t, 4.5). Together with the molecular formula C_29_H_23_NO_6_, the quaternary carbon signal at δ_C_ 120.33 in the ^13^C-NMR data (Figure S21, Table 2) indicates the presence of a cyano group in the structure of **7**. In combination with the ^13^C-NMR spectral data and HMBC correlations (Figure S24, Figure 6), these data suggest that **7** should be an 7-epimer of **6**.

However, several exceptions in the ^1^H-NMR spectrum and a slight change in the chemical shift and multiplicity of certain signals were observed (Table 2). Owing to an anisotropic effect of the aromatic ring, the signals at δ_H_ 7.06 (2H, d, 8.5) for H-2(6)a shifted slightly downfield whereas the signals at δ_H_ 5.95 (1H, brs) for H-14a shifted upfield in comparison with the spectrum of **6**. Besides, observation of the downfield shift of H-10(14)b [δ_H_ 6.07 (2H, d, 2.0)] and H-8b [δ_H_ 3.58 (1H, dd, 8.1, 4.5)] further supports the reversed position of the cyano group and ring A1 at C-7 compared with **6**.

The relative stereochemistry of **7** could be determined by analyzing the NOESY spectrum (Figure S25, Figure 6), in which the interactions between H-8a and H-7b, H-10(14)b suggest a *cis* configuration between H-8a, H-7b and ring B2. Together with the downfield shift of H-10(14)b and upfield shift of H-14a in comparison to those of **6**, the interaction between H-14a and H-2(6)a suggests that ring A1 must be located near ring A2, whereas the interaction between H-7a and H-8b indicates that H-7a and H-8b are situated in a *cis*-orientation. Therefore, the structure of **7** is characterized as shown in Figure 1.

**Table 2:** ^1^H- and ^13^C-NMR spectroscopic data of **6** and 7 *.

Position	6		7	
	δ_C_	δ_H_	δ_C_	δ_H_
1a	125.89s		125.85s	
2(6)a	129.31d	6.89 (2H, d, 8.5)	130.72d	7.06 (2H, d, 8.5)
3(5)a	115.55d	6.79 (2H, d, 8.5)	116.09d	6.74 (2H, d, 8.5)
4a	157.12s		158.03s	
7a	41.16d	3.76 (1H, d, 8.5)	42.09d	3.96 (1H, d, 8.1)
8a	59.35d	3.01 (1H, t, 4.0)	61.31d	3.26 (1H, t, 4.5)
9a	147.44s		148.66s	
10a	121.28s		122.54s	
11a	154.59s		155.23s	
12a	102.39d	6.38 (1H, d, 2.0)	103.18d	6.30 (1H, d, 1.6)
13a	158.49s		159.02s	
14a	103.90d	6.57 (1H, d, 2.0)	104.72d	5.95 (1H, brs)
1b	135.50s		136.82s	
2(6)b	128.40d	6. 87 (2H, d, 8.5)	129.21d	6.84 (2H, d, 8.5)
3(5)b	114.97d	6.76 (2H, d, 8.5)	115.81d	6.71 (2H, d, 8.5)
4b	155.72s		156.51s	
7b	53.84d	4.28 (1H, d, 4.0)	55.89d	4.27 (1H, d, 4.5)
8b	56.70d	3.52 (1H, dd, 8.5, 4.0)	58.07d	3.58 (1H, dd, 8.1, 4.5)
9b	145.39s		145.53s	
10(14)b	105.13d	5.96 (2H, d, 2.0)	106.10d	6.07 (2H, d, 2.0)
11(13)b	158.49s		159.31s	
12b	100.75d	6.17 (1H, t, 2.0)	101.42d	6.15 (1H, t, 2.0)
CN	120.33s		121.73s	

* Data (δ) were measured in CD_3_COCD_3_ for ^1^H at 500 MHz, and for ^13^C at 125 MHz; The assignments were based on DEPT, HSQC, HMBC, and NOESY experiments, respectively.

Known compounds **3**, **5**, **8**, **9**, **10**, and **11** were identified as shegansu B [20], gnetuhainin I [17], leachianol G [21], leachianol F [21], pallidol [22], and resveratrol *trans*-dehydrodimer [23,24] by comparison of their physical and spectroscopic data with those reported in the literature. Product **5**, which possesses an indane skeleton, was obtained for the first time by direct transformation from isorhapontigenin, and all these products would be rather difficult to obtain by common organic reactions. The transformation catalyzed by hexacyanoferrate (III)/sodium acetate was presumed to occur on the basis of a radical reaction. As a result, the obtained dimers should be racemates, which is consistent with the zero values of their optical rotations.

### 2.4. Discussion of the Probable Coupling Reaction Mechanism

**Scheme 1:**
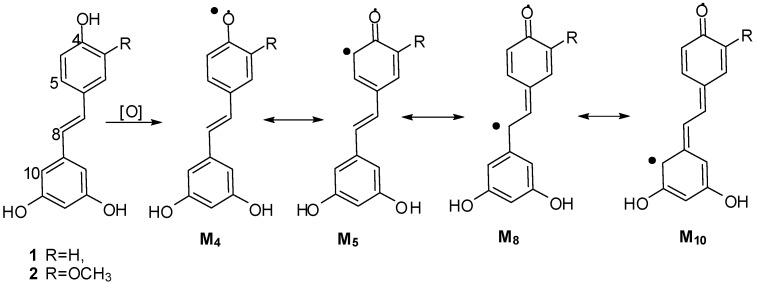
Plausible radicals of **1** and **2**.

On the basis of the aforementioned structures, the dimerization catalyzed by K_3_Fe(CN)_6_/NaOAc was presumed to be based on a radical reaction, induced by K_3_Fe(CN)_6_, whereby stilbene monomers **1** and **2** were dehydrogenated and rearranged to yield radicals M_4_, M_5_, M_8_ and M_10_ (Scheme 1) [11,12,13,14,25,26].

**Scheme 2:**
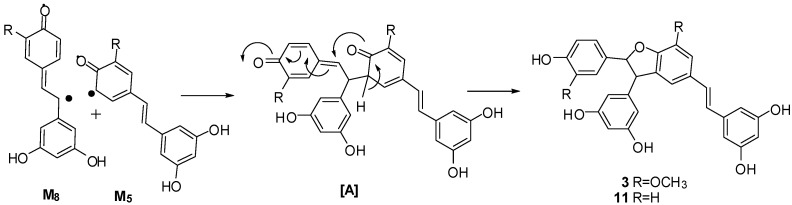
Proposed coupling mechanism of compounds **3** and **11**.

**Scheme 3:**
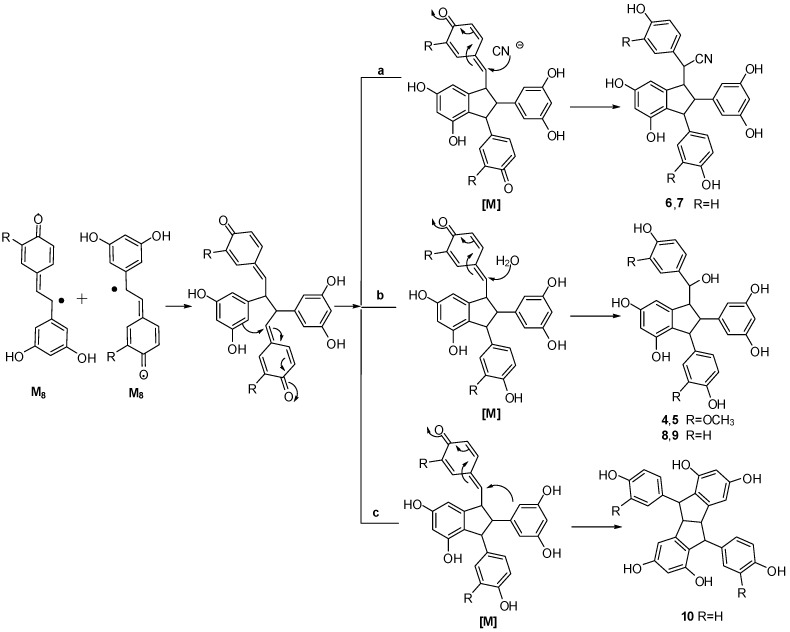
Proposed coupling mechanism of compounds **4**–**10**.

The coupling of radicals M_8_ and M_5_ then occurred successively, followed by tautomeric rearrangement and intramolecular nucleophilic attack to the intermediate quinone [A], yielding the dihydrofunan dimers **3** and **11**, respectively (Scheme 2). Meanwhile, the coupling of two M_8_ radicals, followed by intramolecular cyclization, generates intermediate quinone [M]. The difference in the ultimate products is apparently due to the difference in the position and reagent of the nucleophilic attack (Scheme 3). In the case of path **a**, intermolecular nucleophilic attack to the intermediate quinone [M] of a cyano anion (CN^−^) produced **7** and its isomer **6**. In the case of path **b**, intermolecular nucleophilic attack to the intermediate quinone of a water molecule produced **4**, **8** and their isomers **5**, **9**. In the case of path c, the second intramolecular nucleophilic attack to the intermediate quinone [M] yielded **10**. Evidently, all reactions mentioned above should be carried out concertedly. In general, free radical reaction is not stereoselective. In the course of nucleophilic attack reaction, the chances of attack to *Re* or *Si* face of intermediates are equal. Therefore, all products are found to be enantiomer pairs.

Moreover, the comparatively high yield of **6** and **7** results from the high nucleophilicity of the cyano anion, and the low yield of **10** results from the low nucleophilicity of the phenyl group. The dimeric structures indicate that, during the long reaction time, the oxidation reactions of hydroxystilbene through K_3_Fe(CN)_6_/NaOAc in aqueous acetone, mainly generate M_5_ and M_8_. Product coupling involving M_4_ and M_10_ was not detected in the course of the reaction. The sterically less hindered radical M_5_ would be easily involved in the reaction, possibly accounting for the higher yield of benzofuran products **3** and **11**. However, an appropriate account for the distinction of reactivity cannot be proposed based only on this evidence.

## 3. Experimental Section

### 3.1. Materials and Instrumentation

Optical rotations were measured on a P2000 polarimeter (JASCO, Tokyo, Japan). UV spectra were obtained on a JASCO P650 spectrometer. IR spectra were recorded on a Nicolet 5700 FT-IR microscope instrument (FT-IR microscope transmission, Thermo Electron Corporation, Madison, WI, USA). 1D and 2D NMR spectra were acquired at 500 or 600 MHz for ^1^H and 125 or 150 MHz for ^13^C, respectively, on INOVA 500 MHz (Varian, Inc., Palo Alto, CA, USA), or Bruker AVANCE III HD 600 MHz spectrometers (Bruker Corporation, Karlsruhe, Germany), in acetone-*d*_6_ or methanol-*d*_4_, with the solvent peaks as references. ESI-MS and HR-ESI-MS data were measured using an AccuToFCS JMST100CS spectrometer (Agilent Technologies, Ltd., Santa Clara, CA, USA). Column chromatography (CC) was performed with silica gel (200–300 mesh, Qingdao Marine Chemical Inc., Qingdao, China). HPLC separation was performed on an instrument consisting of a Waters 515 pump and a Waters 2487 dual λ absorbance detector (Waters Corporation, Milford, MA, USA) with a YMC semi-preparative column (250 mm × 10 mm ID) packed with C18 (5 μM). TLC was carried out with glass precoated silica gel GF254 plates (Qingdao Marine Chemical, Inc.). Spots were visualized under UV light or by spraying with 7% H_2_SO_4_ in 95% EtOH followed by heating.

### 3.2. Treatment of Isorhapontigenin with Potassium Hexacyanoferrate (III)/Sodium Acetate.

To a solution of (*E*)-isorhapontigenin (**2**, 100 mg, 0.388 mmol) in acetone cooled to 0 °C in an ice bath, a mixed solution of K_3_Fe(CN)_6_ (150 mg, 0.4559 mmol) and NaOAc (140 mg, 1.7073 mmol) in 25 mL of water was added under stirring. The reactant was stirred at 0 °C for 1 h under a N_2_ atmosphere, and subsequently stirred for another 15 days at room temperature. The reaction mixture was extracted with ethyl acetate and water, the organic layer was washed with brine, water and dried over anhydrous Na_2_SO_4_ for 24 h. Then it was concentrated *in vacuo* to yield a residue that was subjected to silica gel column chromatography eluting with CHCl_3_–MeOH (10:1, *v*/*v*) to give **3** (52.1 mg, 52.2%) and fraction Fr-1 (14.0 mg). Fr-1 was subsequently subjected to semi-preparative Rp-18 HPLC (column, Rp-18, 250 mm × 10 mm I.D., 5 µm, YMC) eluting with acetonitrile in water (25:75, *v*/*v*) to yield compounds **4** (6.2 mg, 6.0%) and **5** (3.4 mg, 3.3%), respectively.

*Compound* **4**: brown amorphous powder; ${\left[\alpha \right]}_{D}^{20}$ 0 (*c* = 0.66, MeOH); UV (MeOH) λ_max_ (log ε): 229 (sh, 4.47), 281 (4.08) nm; IR (film) *ν*_max_: 3360, 2973, 1605, 1516, 1464, 1344, 1276, 1151, 1127, 1032, 1004, 841, 822, 699 cm^−1^; ^1^H-NMR (600 MHz, CD_3_OD) and ^13^C-NMR (150 MHz, CD_3_OD), see Table 1; ESI-MS *m/z*: 555.3 [M + Na]^+^, 571.2 [M + K]^+^, 531.2 [M − H]^−^, 567.2 [M + Cl]^−^; HR-ESI-MS *m/z*: 531.1662 [M − H]^−^ (cacld. for C_30_H_27_O_9_, 531.1661).

*Gnetuhainin I* (**5**): brown amorphous powder; ${\left[\alpha \right]}_{D}^{20}$ 0 (*c* = 0.70, MeOH); ^1^H-NMR (600 MHz, CD_3_OD) and ^13^C-NMR (150 MHz, CD_3_OD), see Table 1; ESI-MS *m/z*: 555.2 [M + Na]^+^, 571.2 [M + K]^+^, 531.2 [M − H]^−^, 567.2 [M + Cl]^−^; HR-ESI-MS *m/z*: 531.1661 [M − H]^−^ (cacld. for C_30_H_27_O_9_, 531.1661).

### 3.3. Treatment of Resveratrol with Potassium Hexacyanoferrate (III)/Sodium Acetate

To a solution of resveratrol (**1**, 1000 mg) in acetone (100 ml), a mixed solution of K_3_Fe(CN)_6_ (2885.0 mg) and NaOAc (2660.0 mg) in water (125 mL) was added under stirring. The reactant was refluxed for 35 h, after removal of the acetone under reduced pressure, the solution was extracted with ethyl acetate (100 mL × 3), the organic layer was washed with brine, water and dried over anhydrous Na_2_SO_4_ for 24 h. The solution was concentrated *in vacuo* to yield a reaction mixture that was subjected to a silica gel (200–300 mesh, 12.5 g) column and eluted with a gradient of increasing MeOH in CHCl_3_ (15:1, 10:1, 6:1, 4:1, 2:1, 1:1, *v*/*v*) to provide seven fractions, namely, FNR-A~FNR-G. FNR-B (102.1 mg) was unchanged resveratrol, and FNR-C was the main product **11** (187.6 mg, 21.1%). 45 mg FNR-D (132.2 mg) was then separated by semi-preparative Rp-HPLC (column, Rp-18, 250 × 10 mm, 5 µm) eluted using methanol/water (35:65, *v*/*v*) to afford **6** (15.8 mg, 4.9%) and **7** (17.7 mg, 5.9%). FNR-F (110.7 mg) was dealt with in the same manner to give **8** (12.1 mg, 1.3%), **9** (19.5 mg, 2.1%) and **10** (7.1 mg, 0.8%), respectively.

*Compound* **6**: a light gray amorphous powder. ${\left[\alpha \right]}_{D}^{20}$ 0 (*c* = 0.33, MeOH); UV (MeOH) λ_max_ (log ε): 206 (4.48), 228 (4.21, sh), 282 (3.59) nm; IR (film) *ν*_max_: 3393.9, 2920.3, 2849.7, 2248.6, 1610.6, 1513.2, 1468.4, 1344.1, 1247.6, 1157.3, 1003.3, 835.7 cm^−1^; ^1^H-NMR (500 MHz in acetone-*d*_6_) and ^13^C-NMR (125 MHz in acetone-*d*_6_), see Table 2; (+)-ESI-MS *m/z*: 482 [M + H]^+^, 504 [M + Na]^+^, 520 [M + K]^+^; HR-ESI-MS *m/z*: 504.1409 [M + Na]^+^ (cacld. for C_29_H_23_NO_6_Na, 504.1418).

*Compound* **7**: a light brown amorphous powder. ${\left[\alpha \right]}_{D}^{20}$ 0 (*c* = 0.66, MeOH); UV (MeOH) λ_max_ (log ε): 204 (4.55), 230 (4.22, sh), 283 (3.70), nm; IR (film) *ν*_max_: 3337.0, 2916.6, 2850.1, 1610.9, 1513.5, 1468.2, 1342.0, 1247.8, 1155.6, 1006.9, 834.0 cm^−1^; ^1^H-NMR (500 MHz in acetone-*d*_6_) and ^13^C-NMR (125 MHz in acetone-*d*_6_), see Table 2; (+)-ESI-MS *m/z*: 482 [M + H]^+^, 504 [M + Na]^+^, 520 [M + K]^+^; HR-ESI-MS *m/z*: 504.1431 [M + Na]^+^ (cacld. for C_29_H_23_NO_6_Na, 504.1418).

*Leachianol G* (**8**): a brown amorphous powder. ${\left[\alpha \right]}_{D}^{20}$ 0 (*c* = 1.05, MeOH); ^1^H-NMR (500 MHz in acetone-*d*_6_) data: 7.07 (2H, d, 8.5, H-2(6)a), 6.73 (2H, d, 8.5, H-3(5)a), 4.45 (1H, d, 8.2, H-7a), 3.38 (1H, dd, 8.2, 3.5, H-8a), 6.22 (1H, d, 2.0, H-12a), 5.71 (1H, d, 2.0, H-14a), 6.87 (2H, d, 8.5, H-2(6)b), 6.71 (2H, d, 8.5, H-3(5)b), 4.27 (1H, d, 3.5, H-7b), 3.50 (1H, t, 3.5, H-8b), 6.14 (2H, d,1.5, H-10(14)b), 6.16 (1H, t, 1.5, H-12b). ^13^C-NMR (125 MHz in acetone-*d*_6_): 135.81s (C-1a), 129.48d (C-2(6)a), 115.18d (C-3(5)a), 157.23s (C-4a), 77.25d (C-7a), 62.69d (C-8a), 147.38s (C-9a), 123.04s (C-10a), 154.84s (C-11a), 102.17d (C-12a), 158.49s (C-13a), 105.55d (C-14a), 138.09s (C-1b), 129.31d (C-2(6)b), 115.55d (C-3(5)b), 156.18s (C-4b), 55.93d (C-7b), 59.04d (C-8b), 151.63s (C-9b), 106.07d (C-10(14)b), 159.21s (C-11(13)b), 100.87d (C-12b). (+)-ESI-MS *m/z*: 473 [M + H]^+^, 495 [M + Na]^+^, 511 [M + K]^+^; HR-ESI-MS *m/z*: 495.1419 [M + Na]^+^ (cacld. for C_28_H_24_O_7_Na, 495.1414).

*Leachianol F* (**9**): a brown amorphous powder. ${\left[\alpha \right]}_{D}^{20}$ 0 (*c* = 1.05, MeOH); ^1^H-NMR (500 MHz in acetone-*d*_6_) data: 6. 86 (2H, d, 8.5, H-2(6)a), 6.68 (2H, d, 8.5, H-3(5)a), 4.46 (1H, d, 8.0, H-7a), 3.37 (1H, dd, 8.0, 3.5, H-8a), 6.32 (1H, d, 1.5, H-12a), 6.59 (1H, d , 1.5, H-14a), 6.85 (2H, d, 8.5, H-2(6)b), 6.74 (2H, d, 8.5, H-3(5)b), 4.24 (1H, d, 3.5, H-7b), 2.95 (1H, t, 3.5, H-8b), 5.93 (2H, d, 2.0, H-10(14b), 6.13 (1H, t, 2.0, H-12b), 4.01 (1H, d, 3.5, 7-OH). ^13^C-NMR (125 MHz in acetone-d_6_): 136.28s (C-1a), 128.87d (C-2(6)a), 115.34d (C-3(5)a), 156.26s (C-4a), 76.58d (C-7a), 61.91d (C-8a), 148.84s (C-9a), 122.46s (C-10a), 154.96s (C-11a), 102.19d (C-12a), 158.75s (C-13a), 106.12d (C-14a), 137.46s (C-1b), 129.35d (C-2(6)b), 115.51d (C-3(5)b), 157.06s (C-4b), 55.61d (C-7b), 59.47d (C-8b), 150.75s (C-9b), 105.90d (C-10(14)b), 159.14s (C-11(13)b), 100.99d (C-12b). (+)-ESI-MS *m/z*: 472 [M]^+^, 495 [M + Na]^+^, 511 [M + K]^+^; HR-ESI-MS *m/z*: 495.1425 [M + Na]^+^ (cacld. for C_28_H_24_O_7_Na, 495.1414).

*Pallidol* (**10**): a brown amorphous powder. ${\left[\alpha \right]}_{D}^{20}$ 0 (*c* = 1.05, MeOH); ^1^H-NMR (500 MHz in acetone-*d*_6_) δ: 6. 99 (2H, d, 8.5, H-2(6)a), 6.71 (2H, d, 8.5, H-3(5)a), 4.58 (1H, s, H-7a), 3.82 (1H, s, H-8a), 6.20 (1H, d, 1.7, H-10a), 6.63 (1H, d, 1.7, H-10a) , 3.32 (1H, s, OH); ^13^C-NMR (125 MHz in acetone-*d*_6_) δ: 137.74s (C-1a), 129.05d (C-2(6)a), 115.74d (C-3(5)a), 159.27s (C-4a), 60.52d (C-7a), 53.97d (C-8a), 123.19 (C-9a), 102.35s (C-10a), 155.24s (C-11a), 103.28d (C-12a), 156.27s (C-13a), 150.53d (C-14a). (+)-ESI-MS *m/z*: 455 [M + H]^+^, 477 [M + Na]^+^, 493 [M + K]^+^. 

*Resveratrol trans-dehydrodimer * (**11**): a brown amorphous powder. ^1^H-NMR (500 MHz in acetone-*d*_6_), δ: 7.24 (2H, d, *J* = 8.5 Hz, H-2a ,6a), 6.83 (2H, d, *J* = 8.5 Hz, H-3a, 5a), 5.44 (1H, d, *J* = 8.0 Hz, H-7a), 4.45 (1H, d, *J* = 8.0 Hz, H-8a), 6.18 (2H, d, *J* = 2.0 Hz, H-10a, 14a), 6.27 (1H, t, *J* = 2.0 Hz, H-12a), 7.25 (1H, brs, H-2b), 6.87 (1H, d, *J* = 8.5 Hz, H-5b), 7.42 (1H, dd, *J* = 8.5 Hz, 2.0, H-6b), 7.04 (1H, d, *J* = 16.5 Hz, H-7b), 6.89 (1H, d, *J* = 16.5 Hz, H-8b), 6.53 (2H, d, *J* = 2.0 Hz, H-10b, 14b), 6.27 (1H, t, *J* = 2.0 Hz, H-12b); ^13^C-NMR (125 MHz in acetone-d_6_) δ: 131.71 (C-1a), 128.57 (C-2a, 6a), 116.08 (C-3a, 5a), 158.33 (C-4a), 94.03 (C-7a), 57.80 (C-8a), 145.21 (C-9a), 107.33 (C-10a), 159.66 (C-11a), 102.24 (C-12a), 159.66 (C-13a), 107.33 (C-14a), 132.10 (C-1b), 123.90 (C-2b), 132.52 (C-3b), 160.60 (C-4b), 110.12 (C-5b), 128.61 (C-6b), 129.09 (C-7b), 127.19 (C-8b), 140.76 (C-9b), 105.63 (C-10b,14b), 159.45 (C-11b,13b), 102.57 (C-12b); (+) ESI *m*/*z*: 455 [M + H] ^+^, 477 [M + Na]^+^, 493 [M + K]^+^; (−) ESI *m/z*: 453 [M − H]^−^, 489 [M + Cl]^−^.

## 4. Conclusions

The oxidative coupling reaction of isorhapontigenin and resveratrol with K_3_Fe(CN)_6_/NaOAc in aqueous acetone as oxidant, in combination with silica gel column chromatography and preparative Rp-HPLC resulted in the isolation of nine stilbene dimers. The structures of the nine dimers were determined on the basis of spectral analysis and chemical properties. Products **4**, **6**, and **7** are new dimers with indane skeletons, product **5** with an indane skeleton was obtained for the first time by direct transformation from isorhapontigenin, and all products would be rather difficult to obtain by common organic reactions.

Results indicated that, under the reaction conditions, the oxidative coupling reaction of two stilbenes yields only two types of stilbene dimers, namely, the benzofuran dimers **3** and **11** with the highest yields, and the indane dimers **4**, **5**, and **6**–**10** in comparatively low yields. Compared with other non-enzymatic oxidants (such as FeCl_3_, Ag_2_O, AgOAc, and so on), this reaction seems to generate only two types of radical intermediates, namely, M_5_ and M_8_, and only two coupling mechanisms occur to form benzofuran and indane stilbene dimers. Thus, the reaction could be used as a convenient method of synthesizing indane stilbene dimers because of its mild conditions, long reaction time and simple products. To the best of our knowledge, up to now, this is the most detailed report on the potassium hexacyanoferrate (III)-sodium acetate catalyzed biomimetic synthesis of stilbene dimers.

## Supplementary Materials

Supplementary materials can be accessed at: http://www.mdpi.com/1420-3049/20/12/19872/s1.
