# Supplementary material for: Potassium Hexacyanoferrate (III)-Catalyzed Dimerization of Hydroxystilbene: Biomimetic Synthesis of Indane Stilbene Dimers
Source: Molecules. 2015 Dec 18;20(12):22662–73. doi: 10.3390/molecules201219872 (PMC6332065; doi:10.3390/molecules201219872)
Supplement: Supplementary file 1 [file molecules-20-19872-s001.pdf]

Supplementary Materials: Potassium Hexacyanoferrate (III)-Catalyzed Dimerization of Hydroxystilbene: Biomimetic Synthesis of Indane Stilbene Dimers

Jing-Shan Xie, Jin Wen, Xian-Fen Wang, Jian-Qiao Zhang, Ji-Fa Zhang, Yu-Long Kang, You-Wei Hui, Wen-Sheng Zheng and Chun-Suo Yao

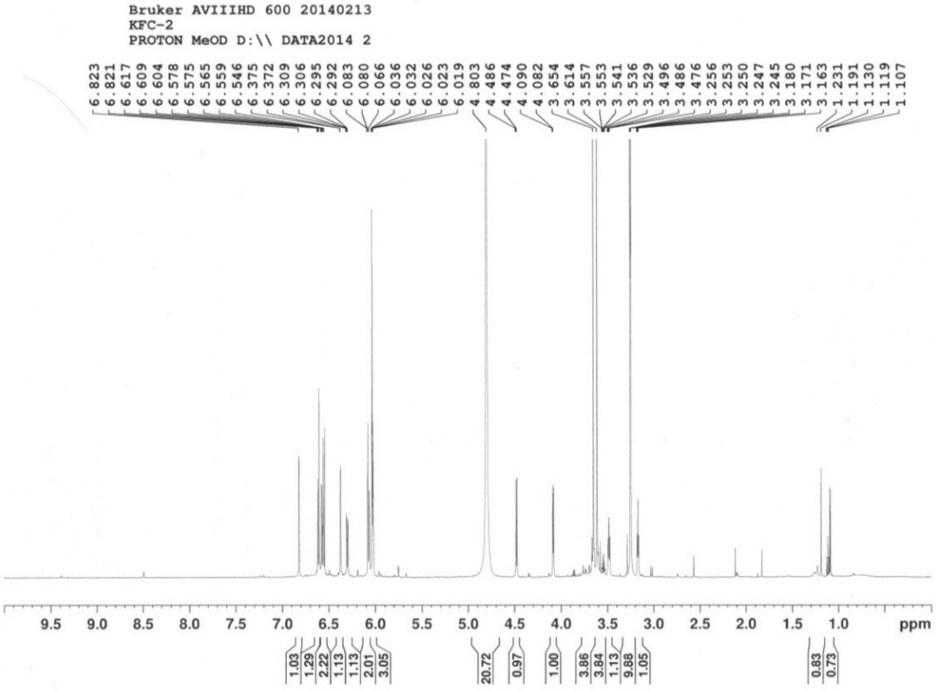

Figure S1. ^{1}H-NMR spectrum of compound (**4**) in CD_3OD.

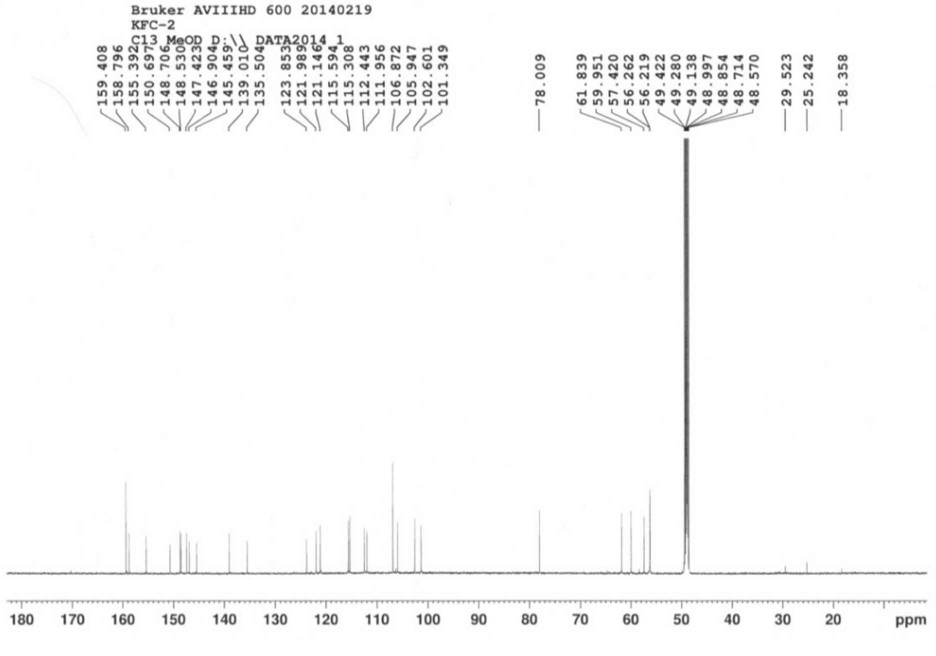

Figure S2. ^{13}C-NMR spectrum of compound (**4**) in CD_3OD.

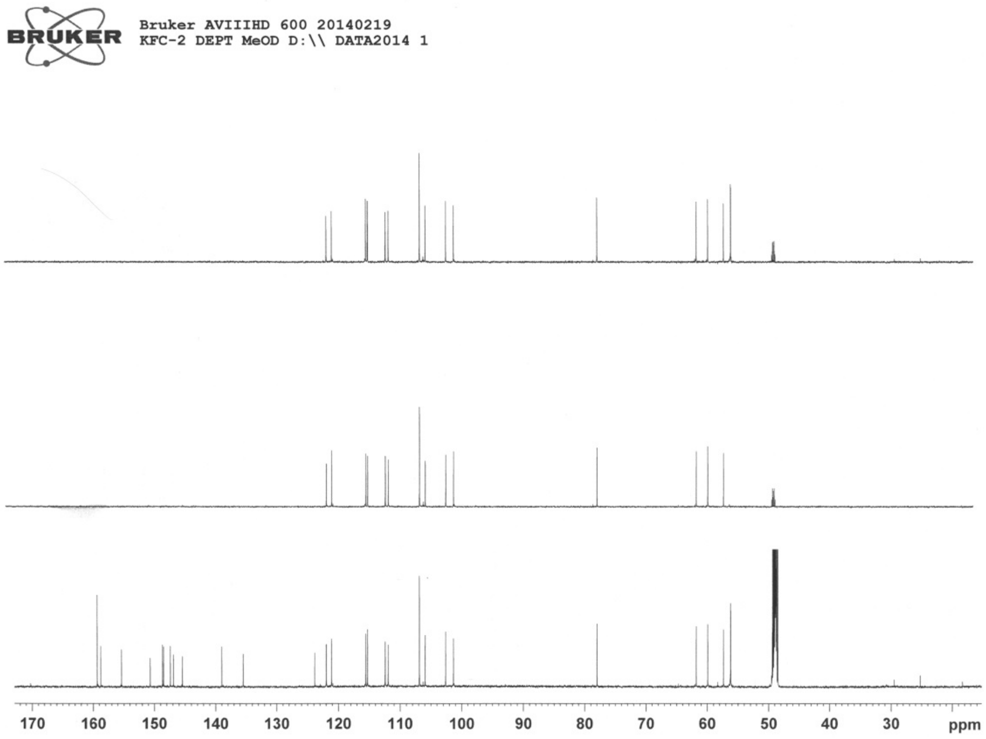

Figure S3. DEPT spectrum of compound (**4**) in CD_3OD.

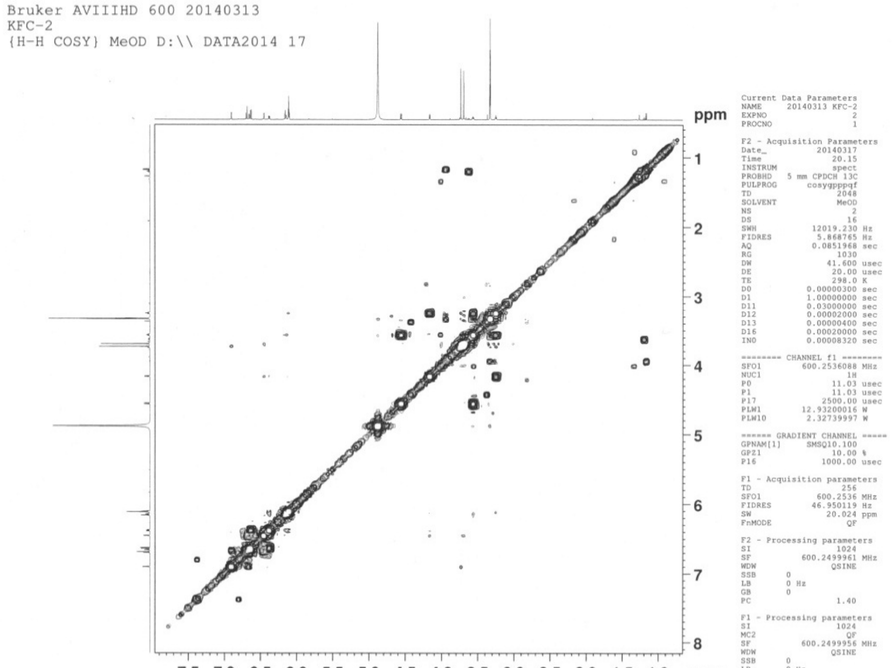

Figure S4. COSY spectrum of compound (**4**) in CD_3OD.

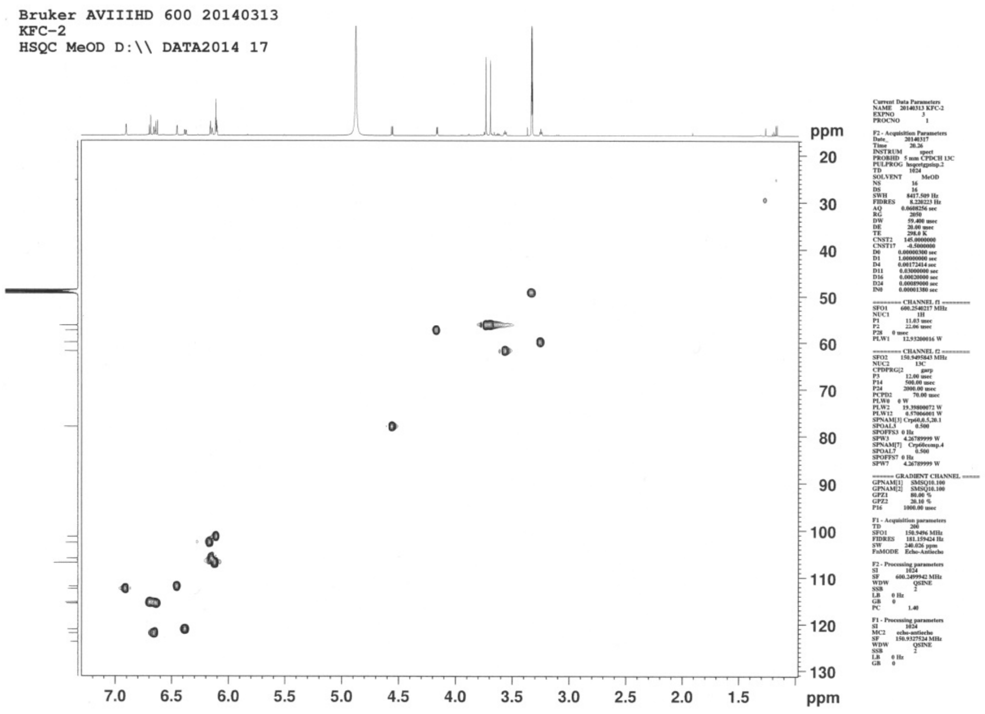

Figure S5. HSQC spectrum of compound (**4**) in CD_3OD.

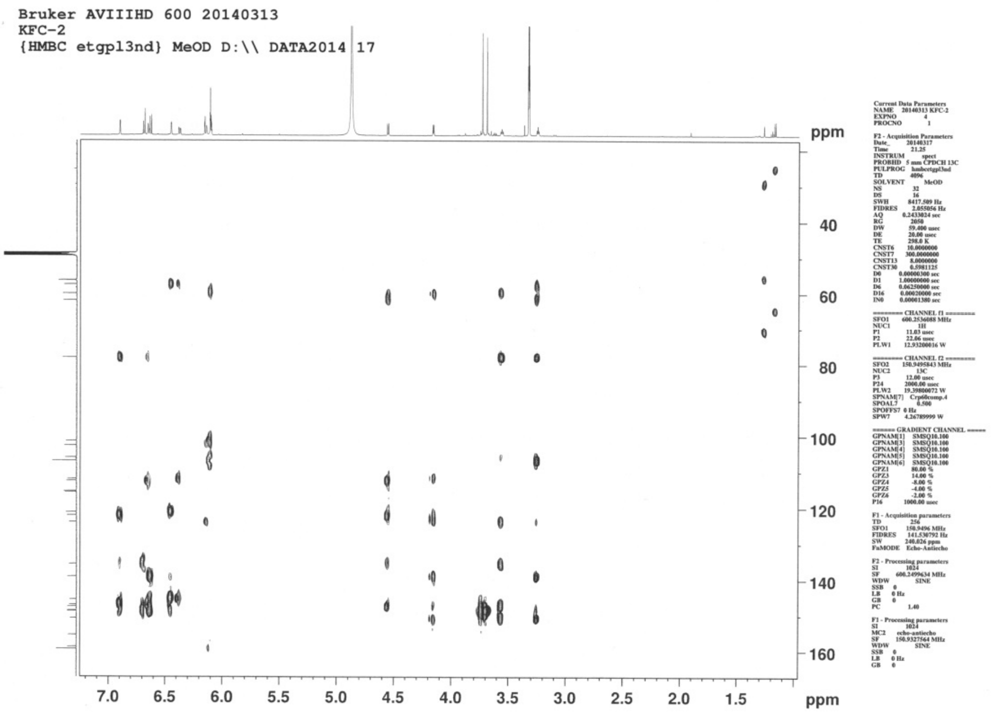

Figure S6. HMBC spectrum of compound (**4**) in CD_3OD.

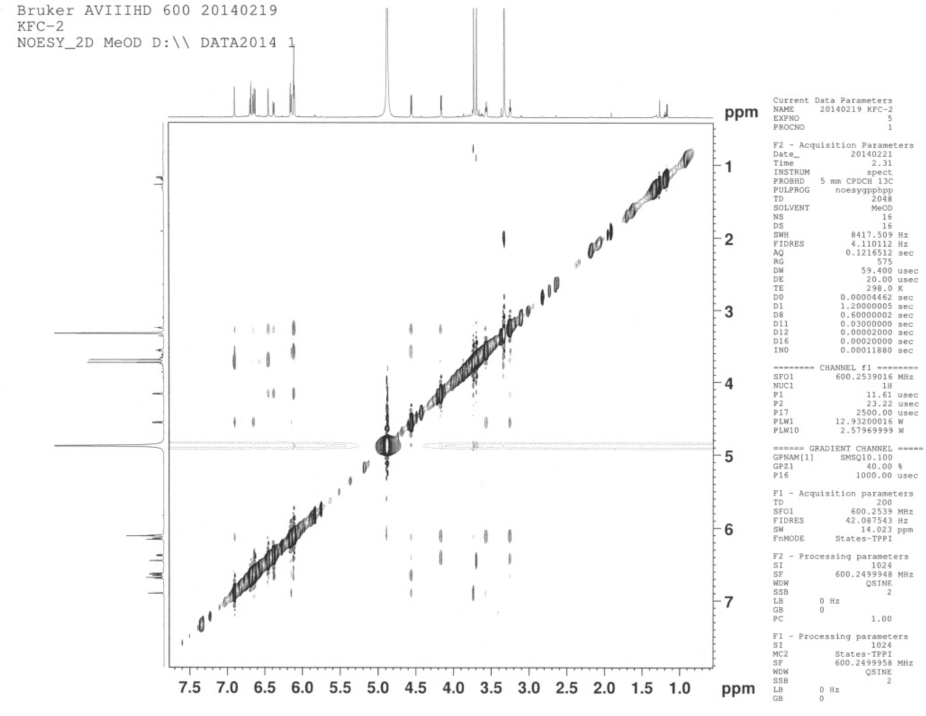

Figure S7. NOESY spectrum of compound (**4**) in CD_3OD.

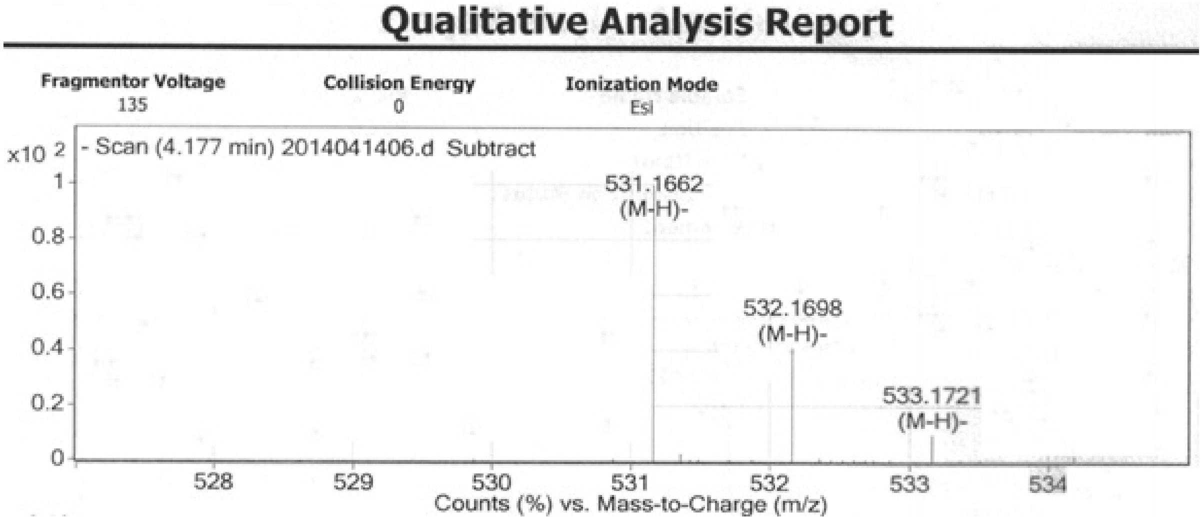

MS Formula Results: - Scan (4.177 min) Sub (2014041406.d)

m/z	Ion	Formula	Abundance
531.1662	(M-H)-	C30 H27 O9	157682.9

Best	Formula (M)	Ion Formula	Calc m/z	Score	Cross S	Mass	Calc Mass	Diff (ppm)	Abs Diff (ppm)	Abund Match	Spacing Mat	Mass Match	m/z	DBE
☐	C31 H24 N4 O5	C31 H23 N4 O5	531.1674	99.42		532.1735	532.1747	2.15	2.15	98.27	99.98	99.84	531.1662	22
☑	C30 H28 O9	C30 H27 O9	531.1661	99.02		532.1735	532.1733	-0.33	0.33	96.58	99.99	100	531.1662	17

Figure S8. HRESIMS spectrum of compound (**4**).

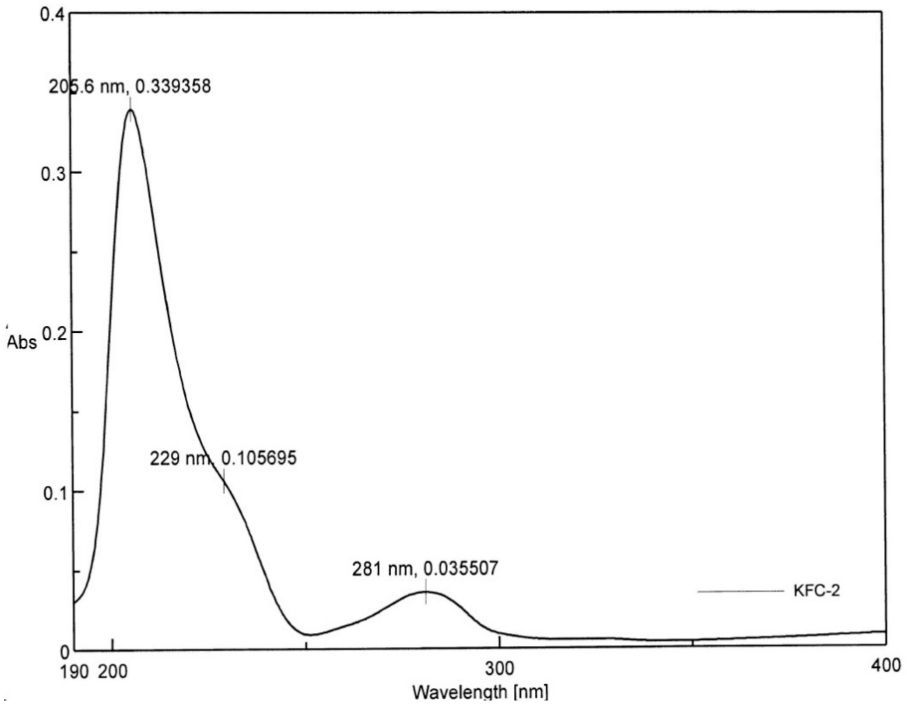

Figure S9. UV spectrum of compound (**4**) in CH_3OH.

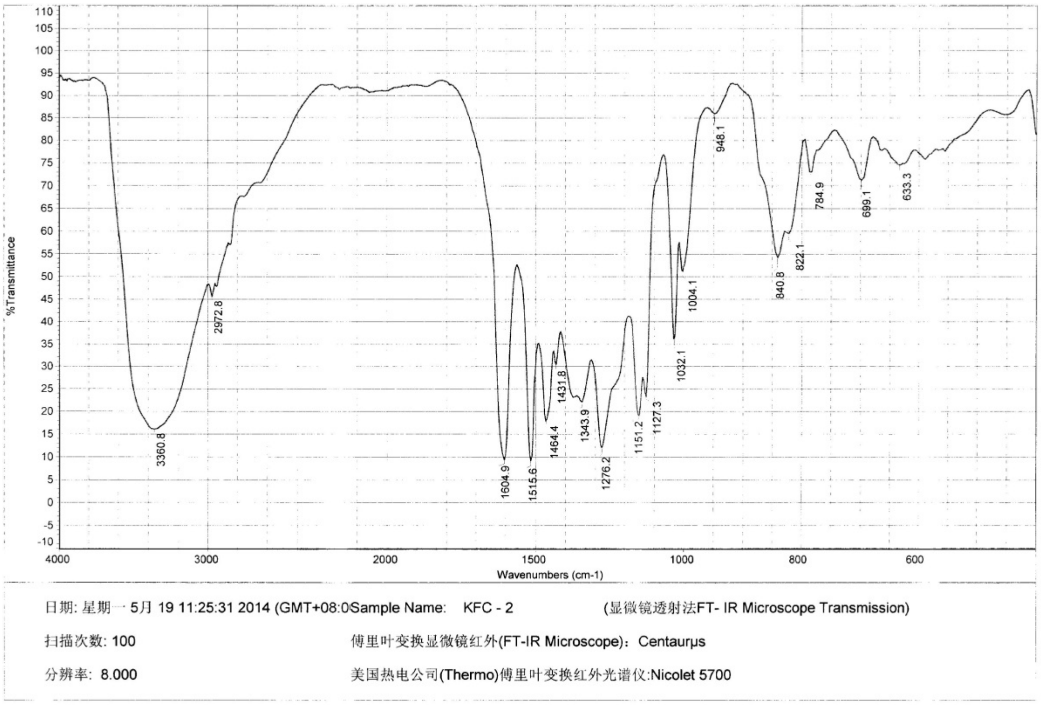

Figure S10. IR spectrum of compound (**4**).

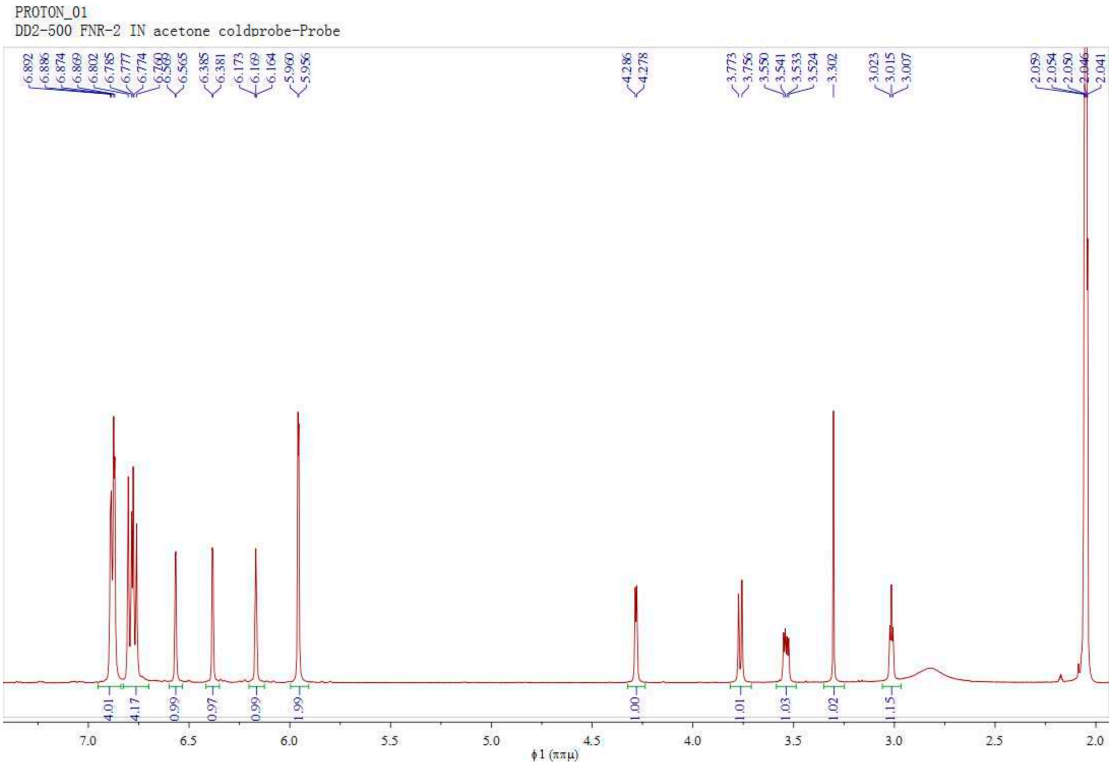

Figure S11. ^1H-NMR spectrum of compound (**6**) in CD_3COCD_3.

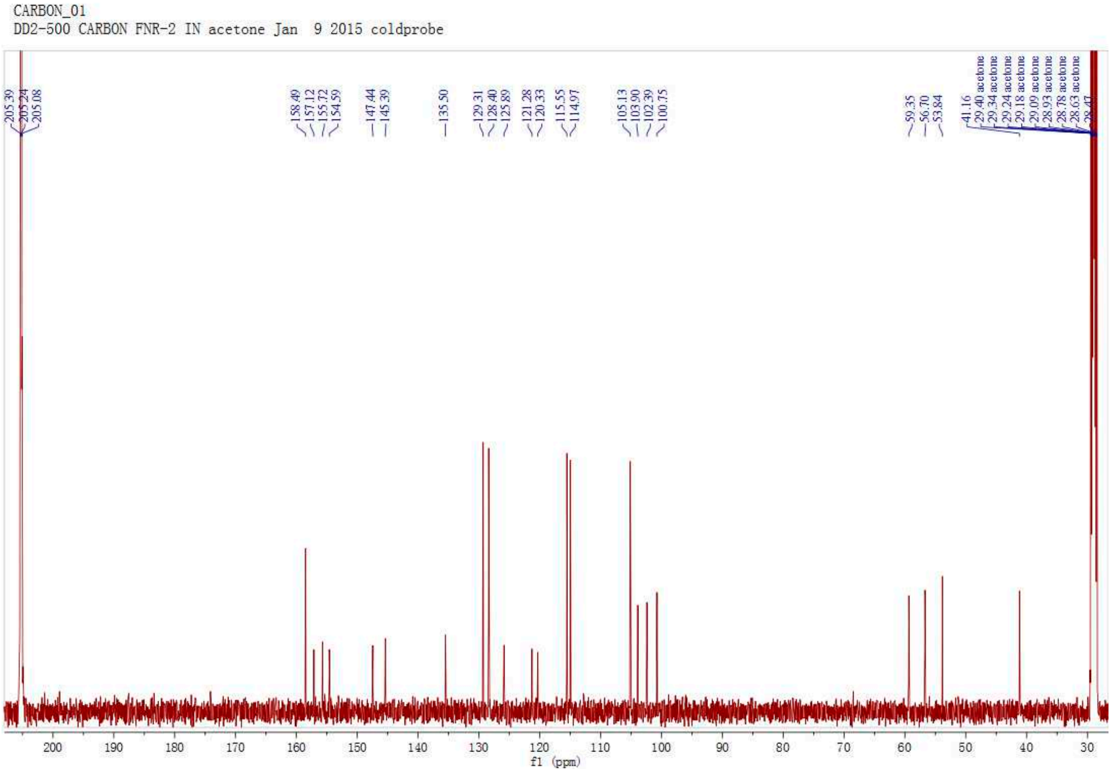

Figure S12. ^{13}C-NMR spectrum of compound (**6**) in CD_3COCD_3.

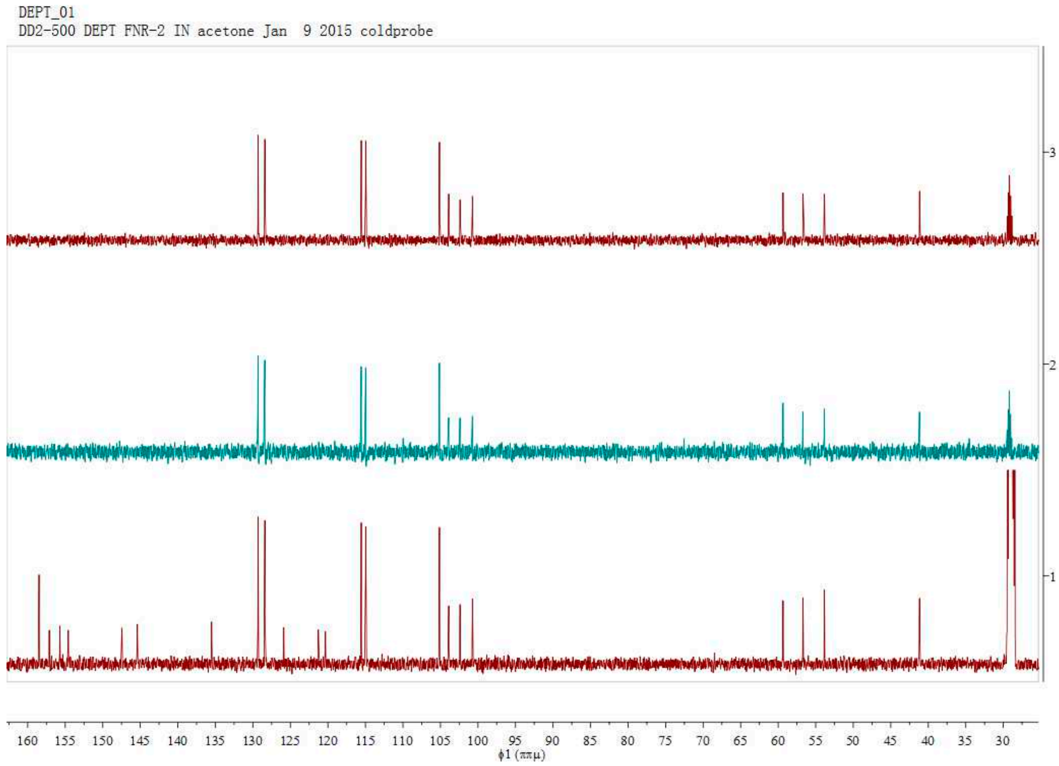

Figure S13. DEPT spectrum of compound (**6**) in CD_3COCD_3.

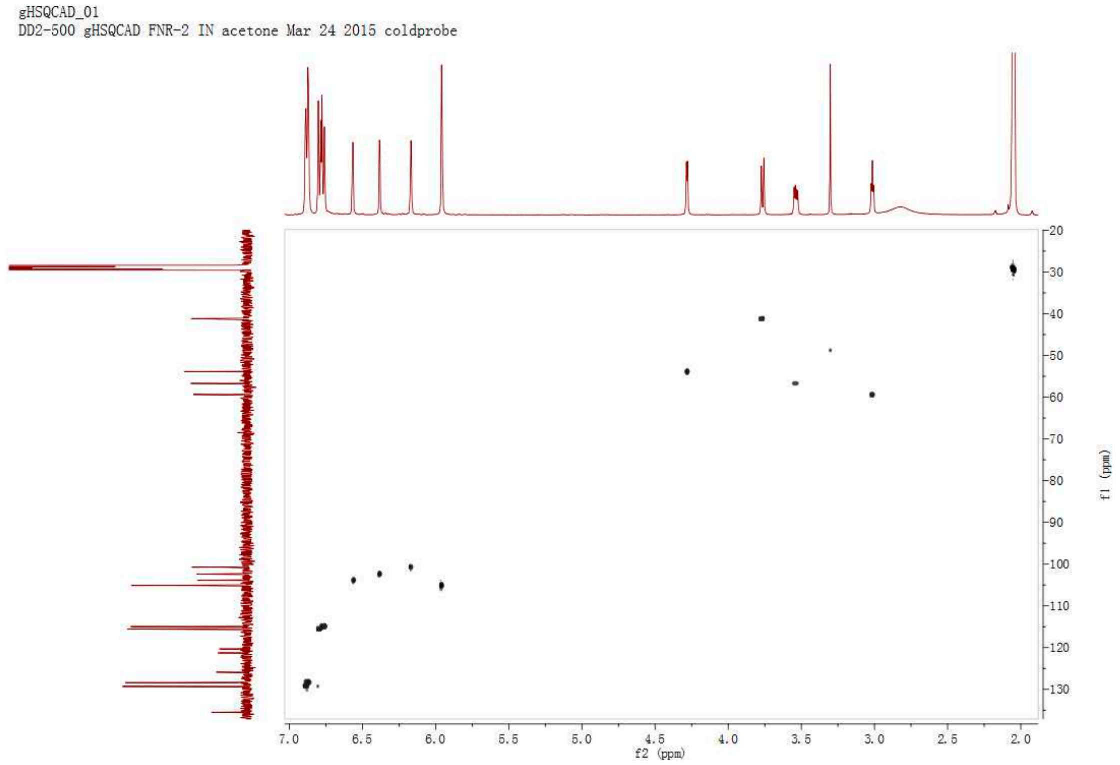

Figure S14. HSQC spectrum of compound (**6**) in CD_3COCD_3.

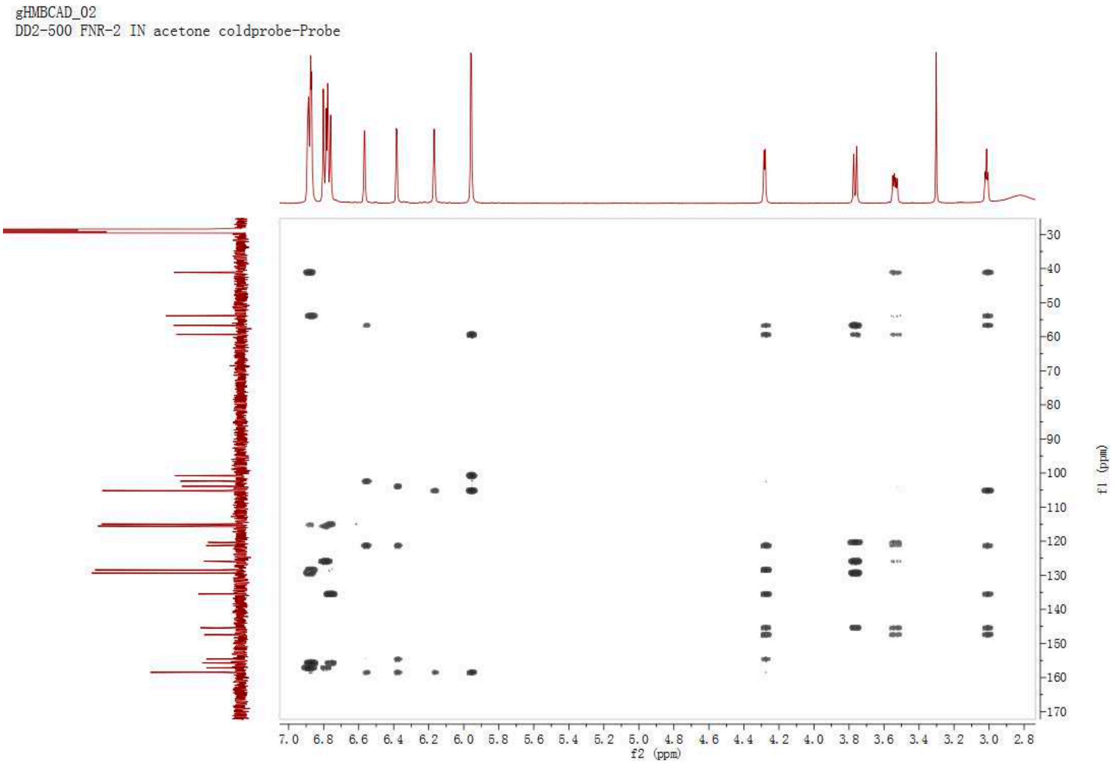

Figure S15. HMBC spectrum of compound (**6**) in CD_3COCD_3.

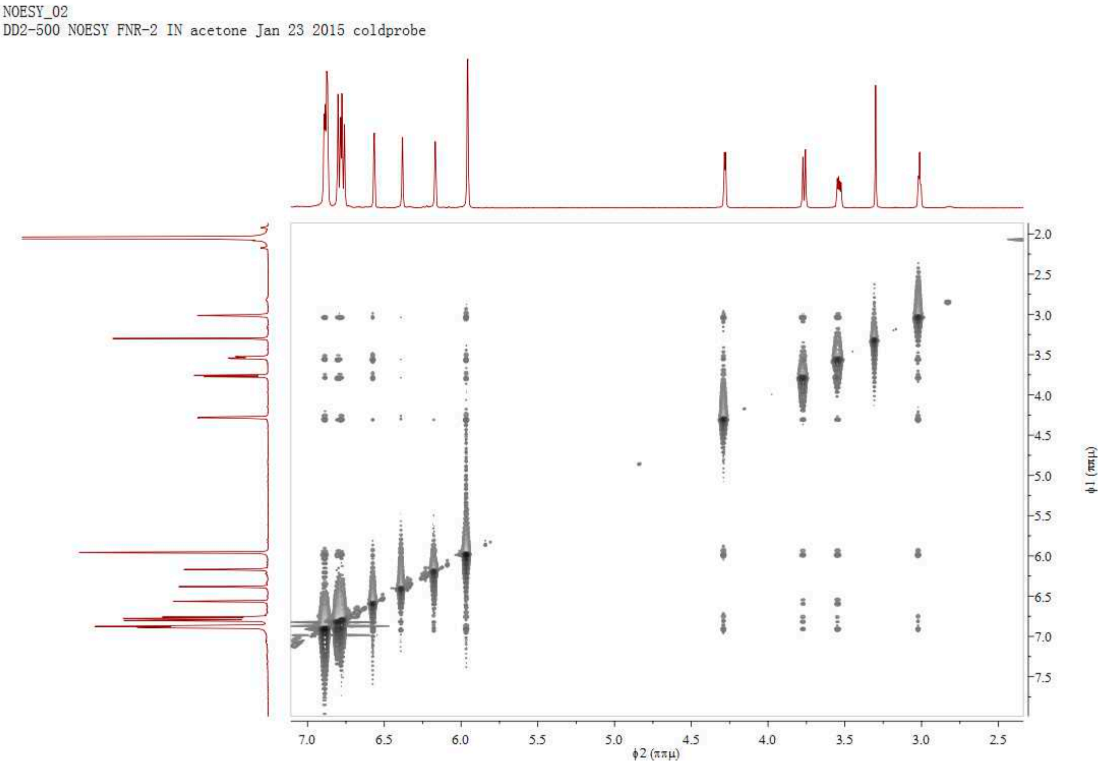

Figure S16. NOESY spectrum of compound (**6**) in CD_3COCD_3.

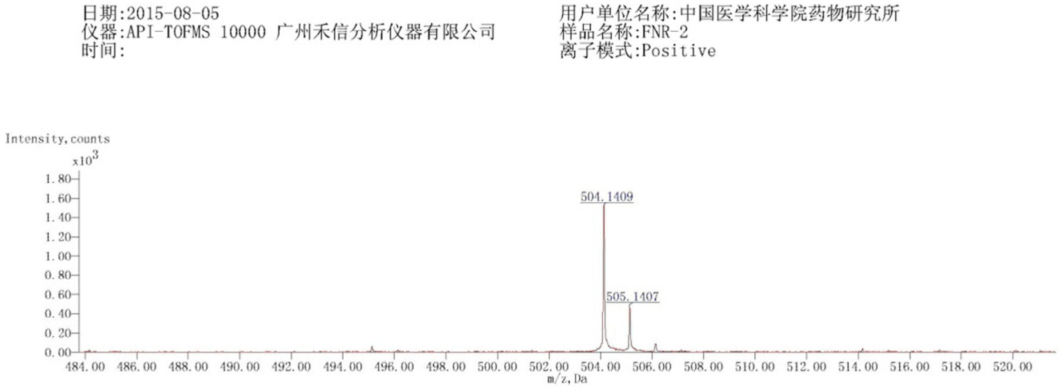

Figure S17. HRESIMS spectrum of compound (**6**).

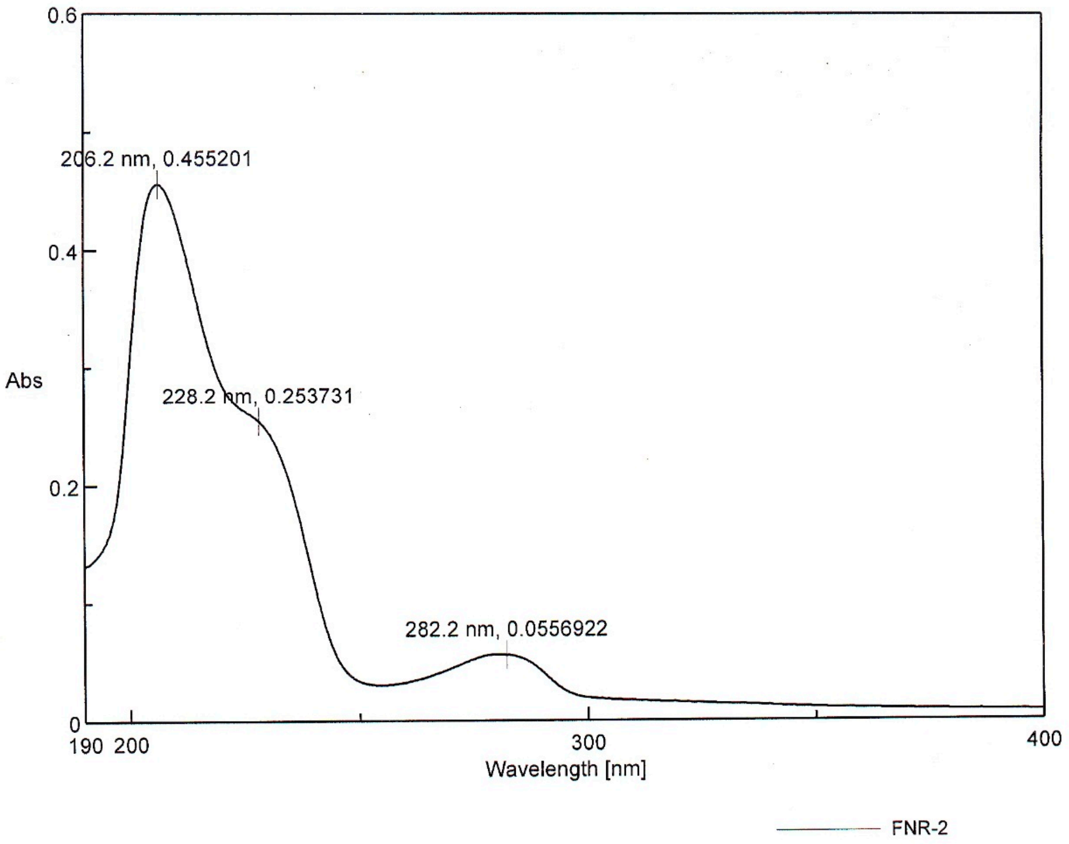

Figure S18. UV spectrum of compound (**6**) in CH_3OH.

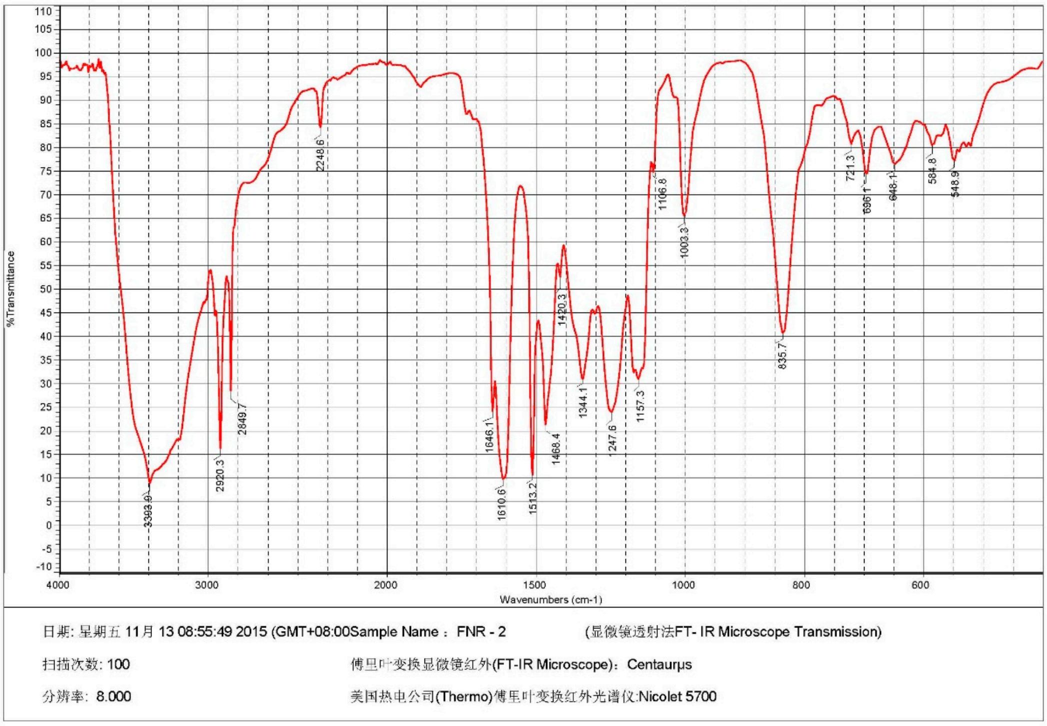

Figure S19. IR spectrum of compound (**6**).

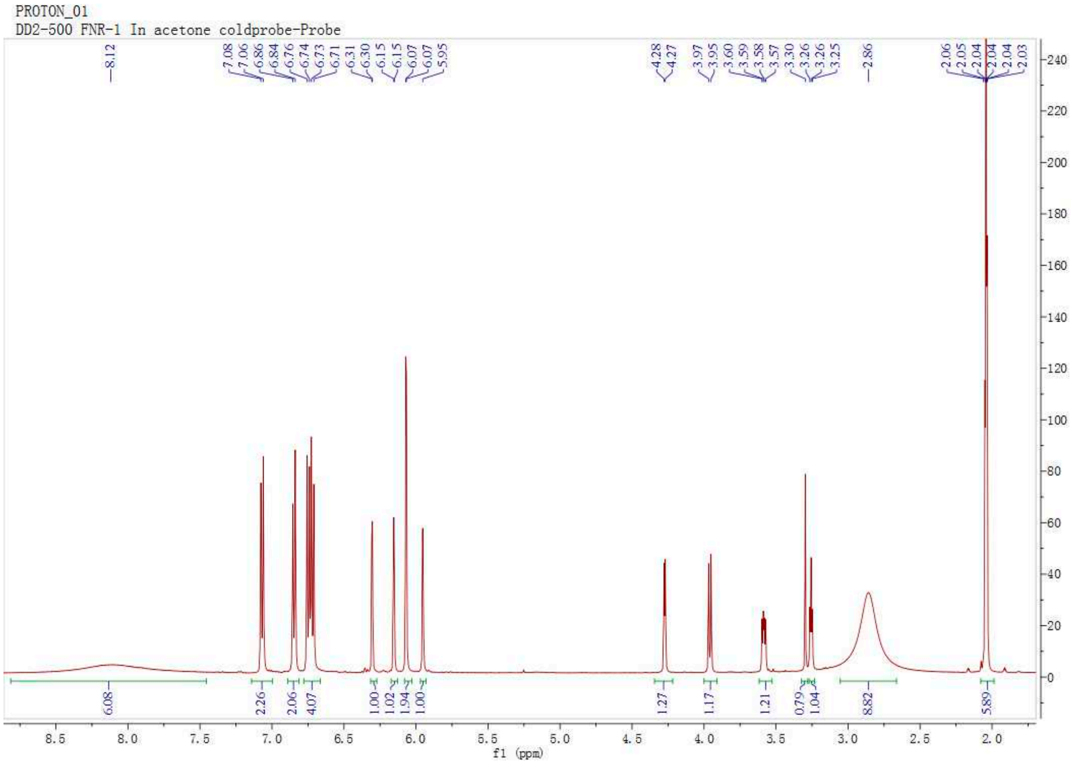

Figure S20. ^{1}H-NMR spectrum of compound (**7**) in CD_3COCD_3.

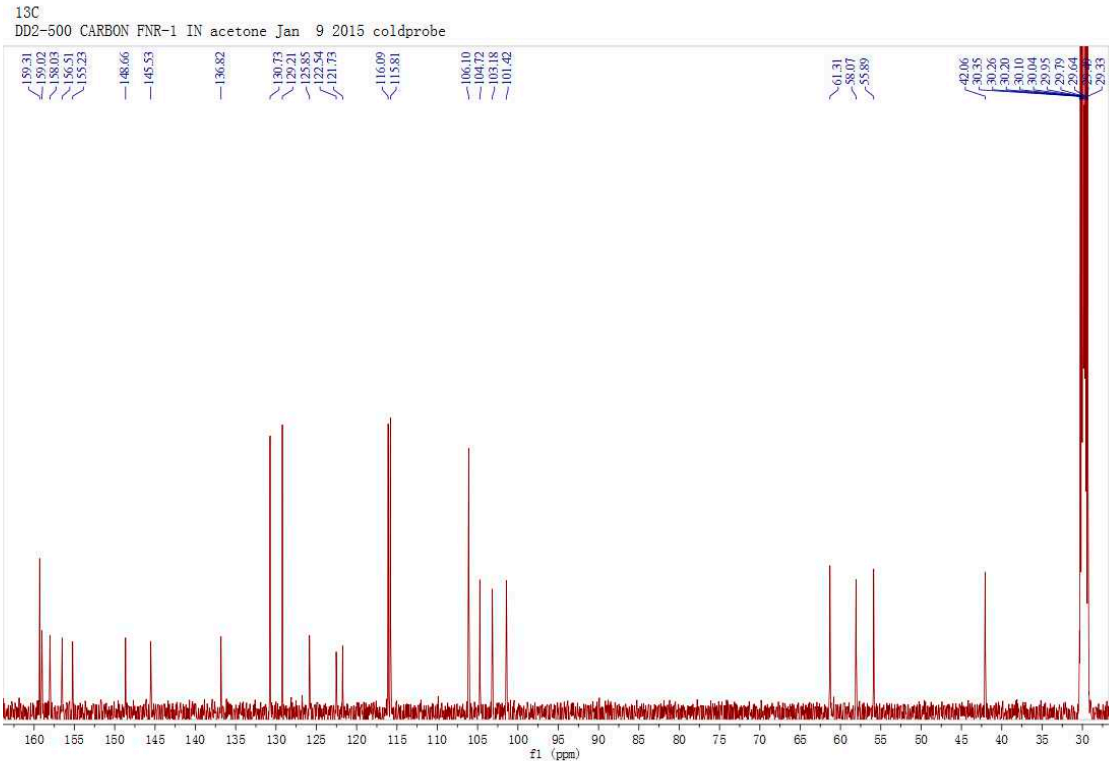

Figure S21. ^{13}C-NMR spectrum of compound (**7**) in CD_3COCD_3.

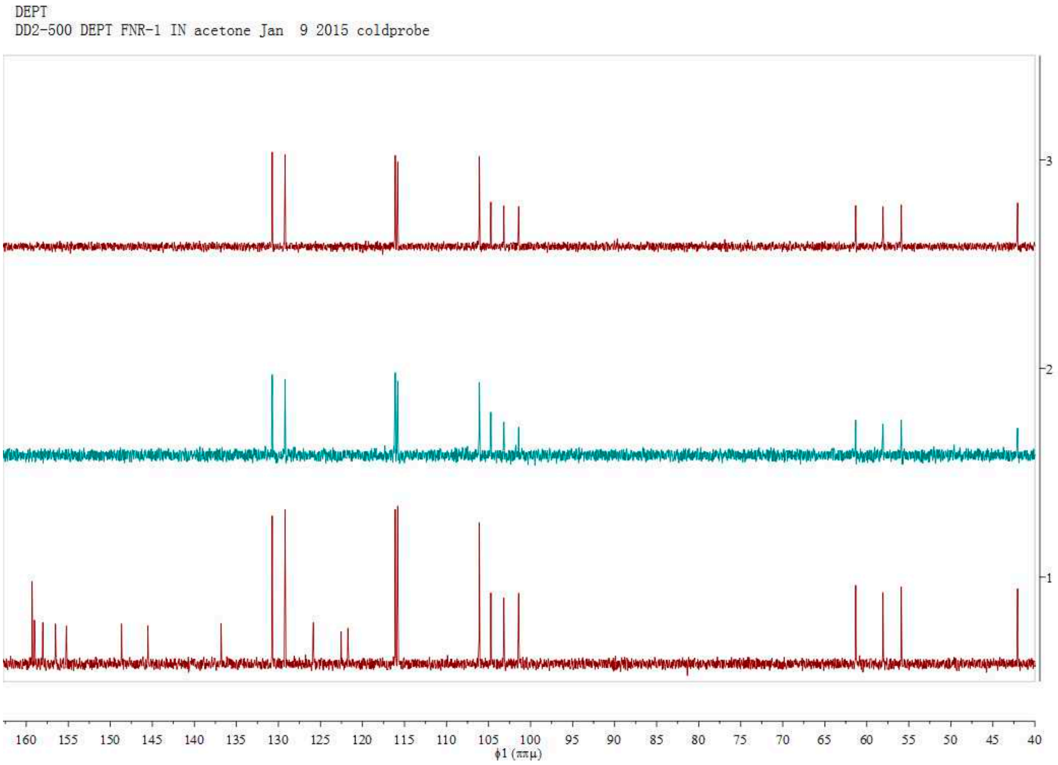

Figure S22. DEPT spectrum of compound (**7**) in CD_3COCD_3.

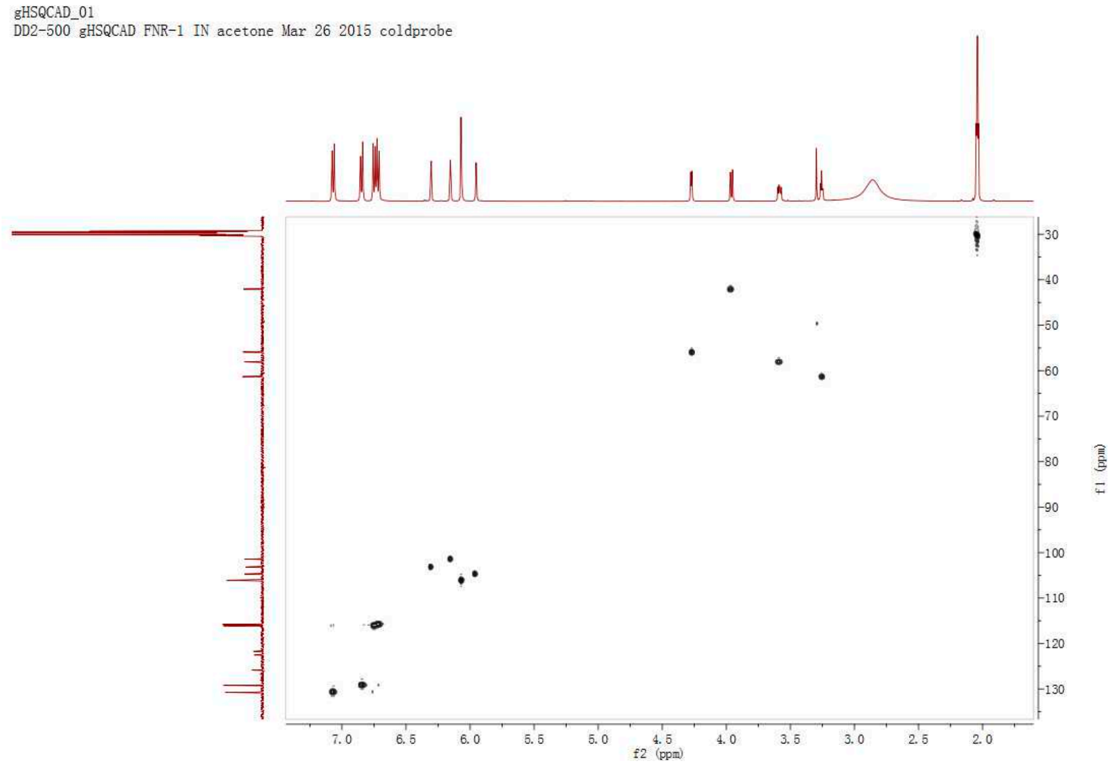

Figure S23. HSQC spectrum of compound (**7**) in CD_3COCD_3.

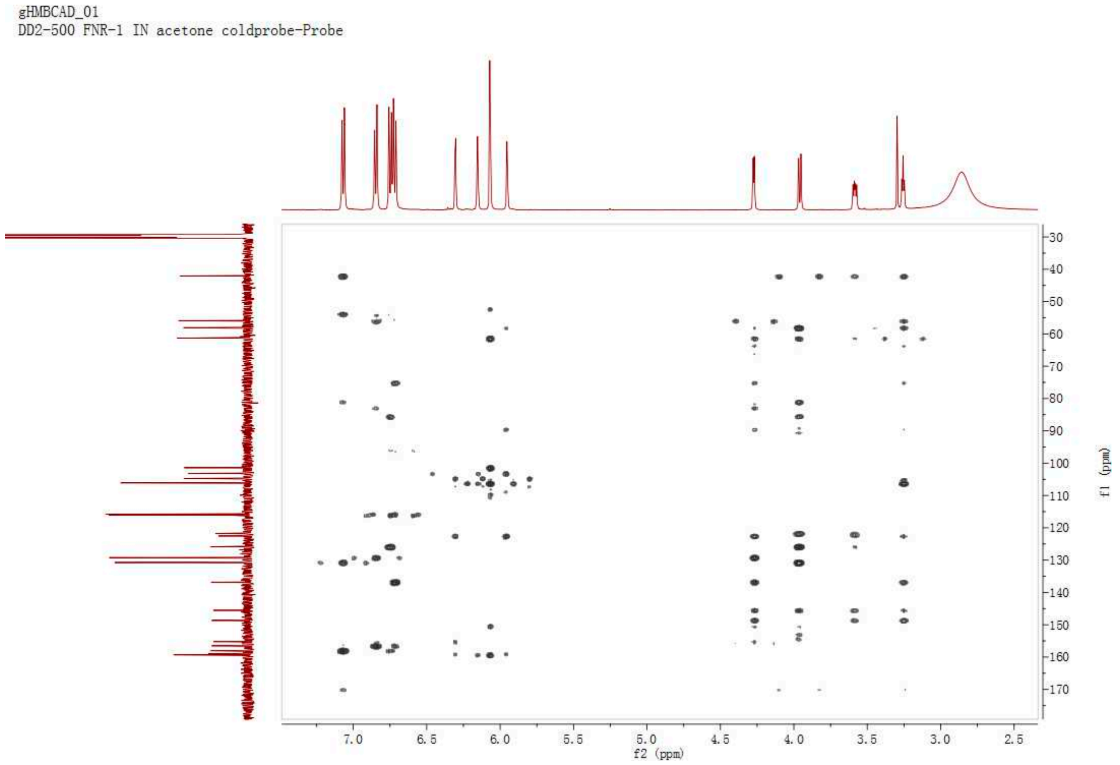

Figure S24. HMBC spectrum of compound (**7**) in CD_3COCD_3.

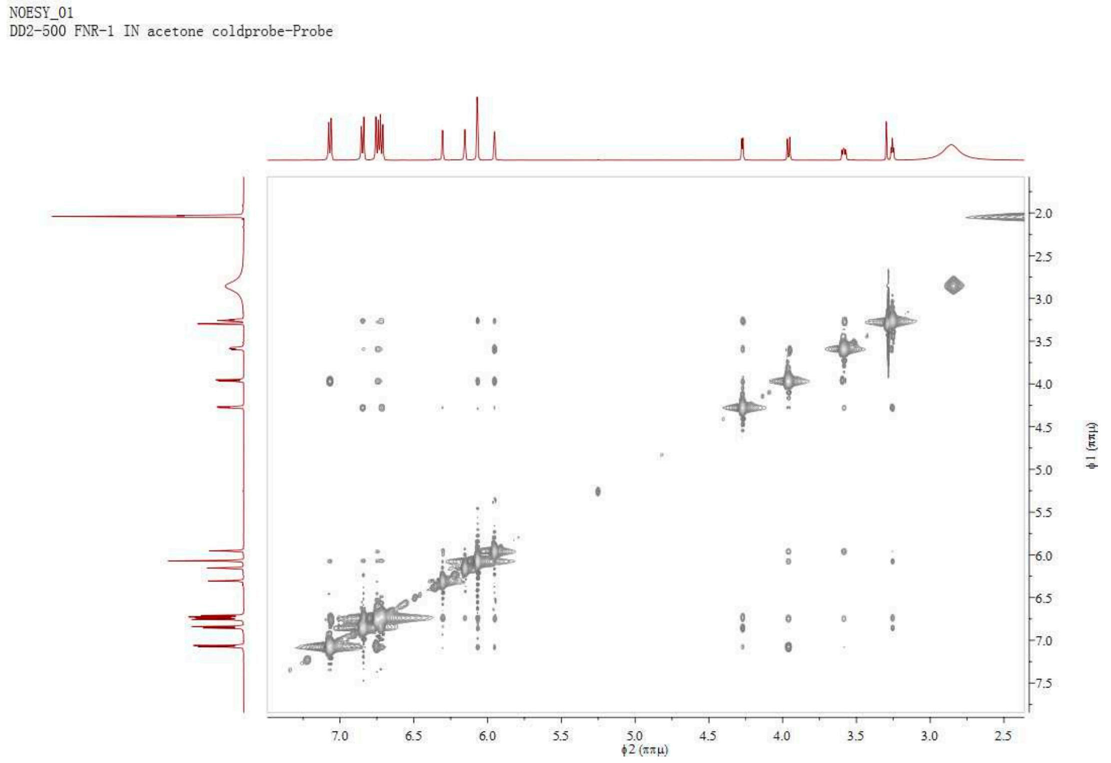

Figure S25. NOESY spectrum of compound (**7**) in CD_3COCD_3.

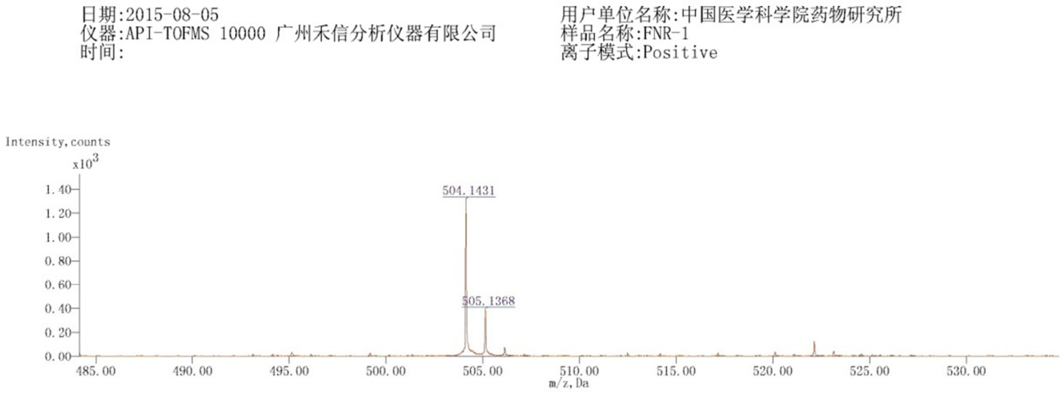

Figure S26. HRESIMS spectrum of compound (**7**).

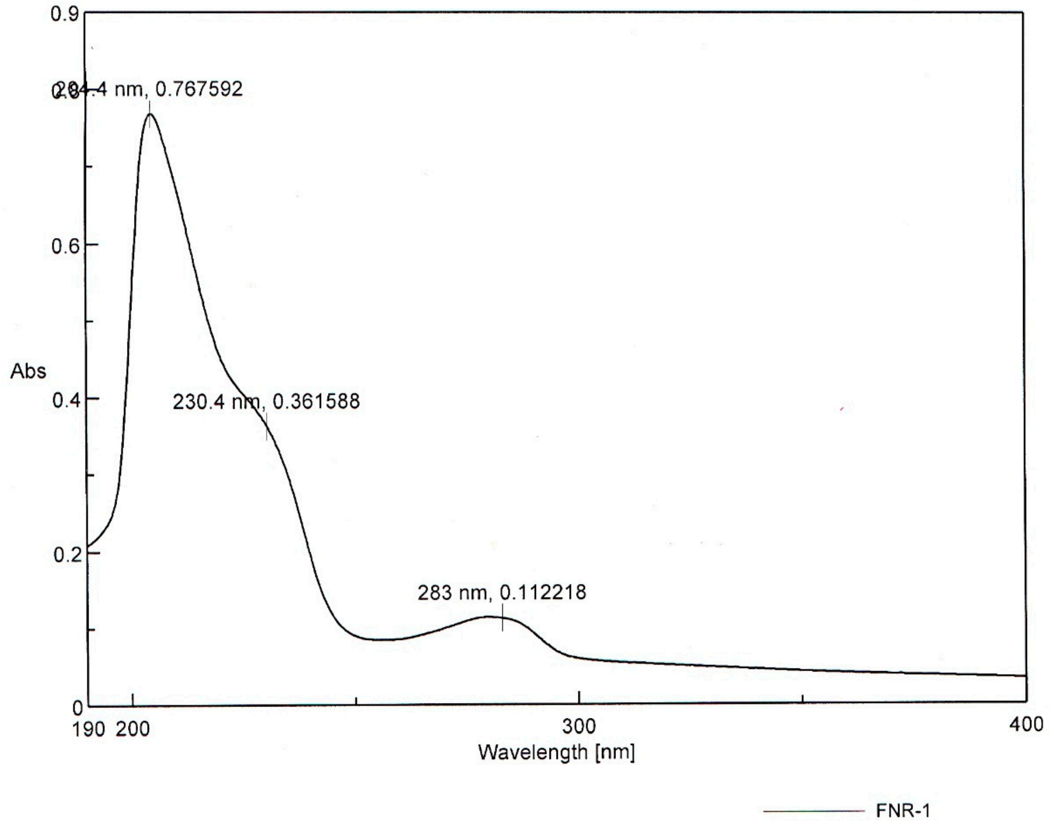

Figure S27. UV spectrum of compound (**7**) in CH_3OH.

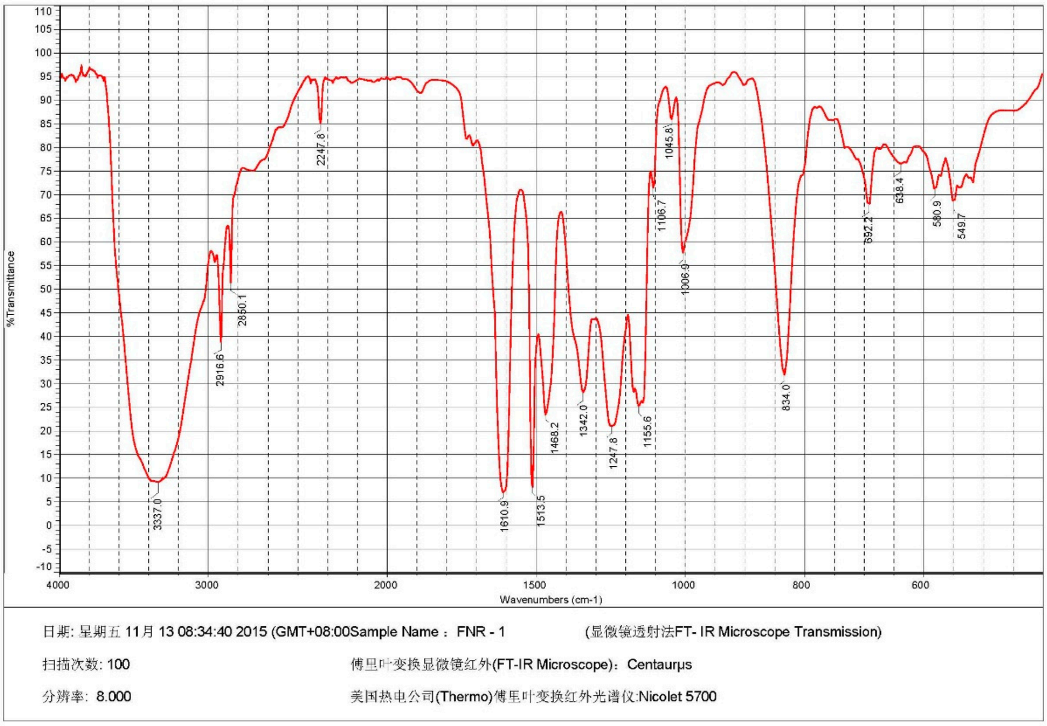

Figure S28. IR spectrum of compound (**7**).
